# Safety and Immunogenicity of Neonatal Pneumococcal Conjugate Vaccination in Papua New Guinean Children: A Randomised Controlled Trial

**DOI:** 10.1371/journal.pone.0056698

**Published:** 2013-02-22

**Authors:** William S. Pomat, Anita H. J. van den Biggelaar, Suparat Phuanukoonnon, Jacinta Francis, Peter Jacoby, Peter M. Siba, Michael P. Alpers, John C. Reeder, Patrick G. Holt, Peter C. Richmond, Deborah Lehmann, for the Neonatal Pneumococcal Conjugate Vaccine Trial Study Team

**Affiliations:** 1 Papua New Guinean Institute of Medical Research, Goroka, Papua New Guinea; 2 Telethon Institute for Child Health Research, Centre for Child Health Research, University of Western Australia, Perth, Western Australia, Australia; 3 Centre for International Health, Curtin University, Perth, Western Australia, Australia; 4 Burnet Institute, Melbourne, Victoria, Australia; 5 School of Paediatrics and Child Health, University of Western Australia, Perth, Western Australia, Australia; ¶ Membership of the Neonatal Pneumococcal Conjugate Vaccine Trial Study Team is provided in Acknowledgments; Menzies School of Health Research, Australia

## Abstract

**Background:**

Approximately 826,000 children, mostly young infants, die annually from invasive pneumococcal disease. A 6-10-14-week schedule of pneumococcal conjugate vaccine (PCV) is efficacious but neonatal PCV may provide earlier protection and better coverage. We conducted an open randomized controlled trial in Papua New Guinea to compare safety, immunogenicity and priming for memory of 7-valent PCV (PCV7) given in a 0-1-2-month (neonatal) schedule with that of the routine 1-2-3-month (infant) schedule.

**Methods:**

We randomized 318 infants at birth to receive PCV7 in the neonatal or infant schedule or no PCV7. All infants received 23-valent pneumococcal polysaccharide vaccine (PPV) at age 9 months. Serotype-specific serum IgG for PCV7 (VT) serotypes and non-VT serotypes 2, 5 and 7F were measured at birth and 2, 3, 4, 9, 10 and 18 months of age. Primary outcomes were geometric mean concentrations (GMCs) and proportions with concentration ≥0.35 µg/ml of VT serotype-specific pneumococcal IgG at age 2 months and one month post-PPV.

**Results:**

We enrolled 101, 105 and 106 infants, respectively, into neonatal, infant and control groups. Despite high background levels of maternally derived antibody, both PCV7 groups had higher GMCs than controls at age 2 months for serotypes 4 (p<0.001) and 9V (p<0.05) and at age 3 months for all VTs except 6B. GMCs for serotypes 4, 9V, 18C and 19F were significantly higher (p<0.001) at age 2 months in the neonatal (one month post-dose2 PCV7) than in the infant group (one month post-dose1 PCV7). PPV induced significantly higher VT antibody responses in PCV7-primed than unprimed infants, with neonatal and infant groups equivalent. High VT and non-VT antibody concentrations generally persisted to age 18 months.

**Conclusions:**

PCV7 is well-tolerated and immunogenic in PNG neonates and young infants and induces immunologic memory to PPV booster at age 9 months with antibody levels maintained to age 18 months.

**Trial Registration:**

ClinicalTrials.gov NCT00219401NCT00219401

## Introduction

Every year an estimated 826,000 children under 5 years of age die of invasive pneumococcal disease (IPD, predominantly pneumonia, meningitis or sepsis), the majority in 3^rd^ world countries [1]. Immunization with 7-valent or 9-valent pneumococcal conjugate vaccine (PCV) starting at 6–8 weeks of age reduces IPD-related morbidity and mortality in vaccinated children and also reduces IPD incidence in unimmunized people through herd immunity [2], [3], [4], [5], [6], [7], [8]. More recently, PCVs with broader serotype coverage (10-valent and 13-valent PCV) have replaced the 7-valent PCV (PCV7). With funding through the Global Alliance for Vaccines and Immunisation (GAVI), PCV has been introduced in 18 developing countries starting at 6 weeks of age and funding has been approved for a further 28 countries [9].

Inclusion of PCVs in routine immunization programs should reduce IPD rates in high-risk populations; however, a significant proportion of disease occurs before children receive PCV due to the early onset of disease and delays in children receiving scheduled vaccines [10], [11], [12]. A multicentre study conducted in children aged <3 months living in 3^rd^ world countries, including Papua New Guinea (PNG), found that the pneumococcus was the most important cause of serious infections in the second and third month of life [13]. In PNG, where the present study was conducted, 26% and 63% of pneumococcal pneumonia in children occurs before ages 2 and 6 months, respectively, and approximately one-third of pneumococcal meningitis occurs before the age of 3 months [14].

Early onset of pneumococcal carriage in the upper respiratory tract (URT) may result in enhanced susceptibility to disease and suboptimal responses to subsequent pneumococcal vaccination [15], [16], [17]. Pneumococcal carriage occurs in very young infants in 3^rd^ world countries: the median age of first pneumococcal acquisition in PNG is 19 days and 24 days in The Gambia [18], [19], [20].

In view of the high rates of disease and carriage in young infants, early interventions such as maternal immunization or accelerated infant immunization schedules, including neonatal vaccination, should be considered in high-risk populations. Neonatal immunization may also improve vaccination coverage in the 3^rd^ world as mothers are more likely to be in contact with health services around the time of delivery. Coverage for Bacillus Calmette-Guérin vaccine (BCG) (recommended at birth) is higher than for vaccines for which a first dose is to be given at age 1 month [10], [11], [21].

In a randomized trial of PCV7 given to Kenyan infants at 6, 10 and 14 weeks or at 0, 10 and 14 weeks with a booster of PCV7 or a fractional dose of 23-valent pneumococcal polysaccharide vaccine (PPV) at 36 weeks of age, neonatal immunization was found to be safe and immunogenic and primed for memory with no evidence of induction of tolerance [22].

In PNG we previously demonstrated efficacy of 23-valent pneumococcal polysaccharide vaccine (PPV) in preventing death and moderate-severe pneumonia when given at 6 months to 5 years of age; serotype-dependent maturation of antibody responses supported efficacy data [23], [24], [25]. A broader range of serotypes causes IPD in high-risk than in low-risk populations [26], [27], [28], [29], which may limit effectiveness of PCV programs. In PNG approximately two-thirds of pneumococcal pneumonia and half of pneumococcal meningitis in children would be covered by a 13-valent PCV [30]. A PPV booster would expand serotype coverage. To address the high burden of pneumococcal disease in young infants living in resource-poor settings, we have conducted an open randomized controlled trial to determine the safety and immunogenicity of PCV7 given either at birth, 1 and 2 months of age (neonatal group) or according to the standard PNG immunization schedule at 1, 2 and 3 months of age with a PPV booster given to all children at age 9 months.

The primary aims of the study were to determine whether:

1. PCV is safe and immunogenic when administered according to the standard immunization schedule in PNG;
2. neonatal immunization with PCV provides earlier antibody responses in infancy than immunization starting at 1 month of age, without compromising safety, especially immunological safety; and
3. neonatal or early infant immunization with PCV induces immunological memory to the relevant pneumococcal polysaccharides.

Here we present the pneumococcal antibody responses and clinical safety data.

## Methods

A detailed description of the study site and population, process of assent and consent, enrolment, immunization and follow-up are reported elsewhere [31]. The protocol for this trial and supporting CONSORT checklist are available as supporting information; see Checklist S1 and Protocol S1. Here we summarize general methods used during the study and present in detail methods pertinent to the results presented here.

### Study Area and Participants

We conducted an open randomized controlled trial in the Asaro Valley in the Eastern Highlands Province of PNG. The valley lies 6° south of the equator and people live at 1500–1900 metres above sea level. The infant mortality rate is 60/1000 live births and 78% of infant deaths occur before the age of 6 months [32]. The PNG Institute of Medical Research (PNGIMR) is located in Goroka town adjacent to the Goroka General Hospital (GGH), which is the only tertiary hospital in the province. Between May 2005 and September 2007 pregnant women were recruited at GGH antenatal clinic and by local reporters in villages located within an hour’s drive of Goroka town. Inclusion criteria for enrolment of newborns were: the intention to remain in the study area for at least 2 years, a birth weight >2000 grams, no acute neonatal infection, no severe congenital abnormality and born in GGH or brought to GGH within 24 hours of birth. Children of mothers known to be HIV-positive were excluded.

### Ethical Considerations

Ethical approval was obtained from the PNG Medical Research Advisory Committee and the Princess Margaret Hospital Ethics Committee in Perth, Australia. This trial is registered at ClinicalTrials.gov under registration number NCT00219401 (http://clinicaltrials.gov/ct2/show/NCT00219401).

Women who had assented were identified by a study nurse around the time of delivery at GGH or if they came to GGH within 24 hours of delivering elsewhere. If still meeting inclusion criteria after clinical examination by a paediatrician, and if the mother provided written consent, the child was enrolled into the study.

An independent data safety monitoring board (DSMB) reviewed all deaths in a timely manner. Other serious adverse events (SAEs, i.e. illness requiring hospitalization) were reported to the independent safety monitor within 48 hours and included in a quarterly progress report for the DSMB.

### Study Procedures and Vaccines

Within 3 days of birth, babies were randomized to receive three doses of PCV7 (Prevnar™, Pfizer) intramuscularly either at birth, 1 and 2 months of age (neonatal group) or at ages 1, 2 and 3 months (infant group), or to receive no PCV7 (control group). Each 0.5 mL dose of PCV7 (batch numbers 15422 and 21933) contains 20 µg of CRM_197_ carrier protein coupled to 2 µg of each saccharide for serotypes 4, 6B, 9V, 14, 18C, 19F, 23F and 0.125 mg aluminium adjuvant. At 9 months of age all children received PPV (batch number G3836) containing 25 µg of purified capsular polysaccharides of each of serotypes 1, 2, 3, 4, 5, 6B, 7F, 8, 9N, 9V, 10A, 11A, 12F, 14, 15B, 17F, 18C, 19F, 19A, 20, 22F, 23F and 33 (Pneumovax 23™, Merck & Co). In addition to the study vaccines, all children received immunizations according to the recommended schedule in PNG at that time [33]: BCG at birth; oral polio vaccine (OPV) at birth, 1, 2 and 3 months; Hepatitis B vaccine (HepB) at birth, 1 and 3 months; a combined diphtheria, tetanus, whole cell pertussis, *Haemophilus influenzae* type b (Hib) vaccine (DTwP/Hib) at ages 1, 2 and 3 months and measles vaccination at 6 and 9 months.

### Randomization

Eligible infants were randomized using a computer-generated list of random numbers [31]. As PCV7 had not been evaluated in this population previously or in a 1-2-3-month schedule, the first 52 infants were randomized to infant and control groups only. Following review of these infants’ safety data, the DSMB approved inclusion of the neonatal arm of the study. At enrolment each child was assigned to neonatal, infant or control group as specified inside a sealed envelope with the next sequential number. Laboratory staff were blinded to the group allocation throughout the study.

### Specimen Collection

Umbilical cord and venous or capillary blood samples at 2, 3, 4, 9, 10 and 18 months of age were collected to separate serum for antibody assays (0.5–2.5 ml). Blood samples were brought to the laboratory within 2 hours of collection. Serum samples were aliquotted and stored at −20°C until they were analysed.

### Pneumococcal Capsular Polysaccharide (CPS) ELISA

Serum serotype-specific IgG to PCV7 serotypes (VT) and to non-PCV7 serotypes (NVT) 2, 5 and 7F which commonly cause IPD in Papua New Guinean children [14], [26] were measured using a WHO standardized pneumococcal enzyme-linked immunosorbent assay (ELISA) [34]. Briefly, microtitre plates (Greiner, Germany) were coated with the specific pneumococcal capsular polysaccharide (CPS) antigens for 5 hours at 37°C. In order to enhance the specificity of the assay by removal of non-specific antibodies, the reference serum 89SF (FDA, Bethesda, MD) was pre-absorbed overnight with 10 µg/ml cell wall polysaccharide (CWPS) and serum samples with 10 µg/ml CWPS and 5 µg/ml purified serotype 22F polysaccharide. Alkaline phosphatase goat anti-human IgG conjugate and *p*nitrophenyl phosphate (Sigma, USA) were used for antibody detection. Each plate contained high and low in-house control sera to assess intra- and inter-assay variations.

### Reactogenicity and Morbidity Surveillance

Children were observed for 1 hour and reactogenicity again assessed 48–96 hours post-vaccination [31]. Morbidity data were collected by passive surveillance at the PNGIMR clinic, routine check of parent-held health books, review of admissions to the GGH paediatric ward, and weekly visits to study participants’ houses by village reporters throughout the first year and fortnightly from age 12–18 months. All children with moderate or severe pneumonia, suspected meningitis or sepsis were referred to a paediatrician. Blood culture and a pernasal swab were to be collected if a child had temperature >38°C or signs of moderate or severe pneumonia (cough, raised respiratory rate and lower chest wall indrawing with or without enlarged liver or cyanosis) or suspected meningitis. Blood culture and characterisation of pneumococcal and *Haemophilus influenzae* isolates were performed according to standard methods established at PNGIMR [14], [26], [35].

### Outcomes of the Study

The primary outcomes for the study were:

1. immunogenicity of PCV7 under an accelerated 1-2-3-month schedule (aim 1) which was assessed by comparing serotype-specific IgG concentrations and the proportions of children with VT serotype-specific antibody concentrations ≥0.35 µg/ml (the serologic correlate of protection against IPD following PCV [36], [37]) at 2, 3 and 4 months of age in infants vaccinated according to the infant schedule with those in infants who had not received PCV7;
2. comparison of serotype-specific IgG concentrations and the proportions of children with serotype-specific antibody concentrations ≥0.35 µg/ml [36], [37]between the neonatal and infant groups at age 2 months for PCV7 serotypes (aim 2);
3. comparison of serotype-specific IgG concentrations and the proportions of children with VT serotype-specific antibody concentrations ≥0.35 µg/ml one month post-PPV between PCV-vaccinated children and controls (who had received PPV) (aim3); and
4. safety of 3 doses of PCV7 in PNG infants when administered according to the standard immunization schedule in PNG (1, 2 and 3 months) and when started at birth (0, 1, and 2 months); rates of local and systemic reactions and hospitalization rates were compared between groups.

Secondary outcomes were VT serotype-specific IgG concentrations and proportions of samples with concentration ≥0.35 µg/ml at ages 3, 4, 9 and 18 months and also serotype-specific IgG concentrations and proportions of responders (≥0.35 µg/ml) for non-PCV7 PPV serotypes 2, 5 and 7F at ages 2, 3, 4, 9, 10 and 18 months.

### Statistical Analyses

Details of sample size calculations have been reported previously [31]. Power analyses conducted during study design showed that a sample size of 100 per vaccination group allows a 70% seroprotection rate (≥0.35 µg/ml*)* to be estimated with a 95% confidence interval of 60% to 79%. This sample size gives 80% power to detect a reduction to 50% from this seroprotection rate as being inferior and an increase to 87% as being superior at a significance level of p<0.05. For the non-inferiority analyses, using a margin of 10%, the sample size has equivalent power to detect a rate of 78% as being non-inferior to 70%.

Per protocol analyses included all children meeting inclusion criteria who received PCV7, PPV and DTP/Hib according to protocol and had appropriate samples collected. Intention-to-treat analysis included all children who met the inclusion criteria for the study. Both intention-to-treat analyses and per protocol analyses were performed and gave similar results. Therefore results of per protocol analyses are presented here.

Data were analysed using SPSS (version 15.0, Chicago, IL, USA). Serotype-specific antibody concentrations were log-transformed and geometric mean concentrations (GMCs) and 95% confidence intervals (CIs) calculated. Exact confidence intervals for binomial proportions were calculated using the Clopper-Pearson method. Categorical variables were compared using the Pearson chi-squared test. The Mann-Whitney or Kruskal-Wallis tests were used to compare continuous data in two or three groups, respectively. We used Pearson chi-squared or Fisher’s exact tests to calculate p-values for group differences in frequency of adverse reactions to vaccination, except where a vaccine was administered on more than one occasion to each child in which cases we used a logistic regression model incorporating general estimating equations to calculate this p-value. Differences were considered significant if the p-value was less than or equal to 0.05.

In addition, a post-hoc superiority analysis was performed by calculating the exact, unconditional, 2-sided 95% CIs on the difference in proportions of children with antibody concentration ≥0.35 µg/ml (considered superior in the neonatal group if the 95% CI does not include 0). If superiority was not achieved, non-inferiority of the neonatal schedule was assessed by determining whether the 2-sided 95% CI for the difference in proportions of children with antibody concentration ≥0.35 µg/ml between the two groups (neonatal–infant) does not exceed −0.10.

Poisson regression was used to adjust age-specific incidence and hospitalization rates (/1000 person-days) for gender. Incidence rate ratios (IRRs) and 95% CIs relative to the control group were used to assess clinical safety of infant and neonatal schedules. Person-time-at-risk was used to determine age-specific hospitalization rates and took into account age at entry (though all were aged <3 days) and exit from the study.

## Results

### Population Characteristics and Follow-up

**Figure 1:**
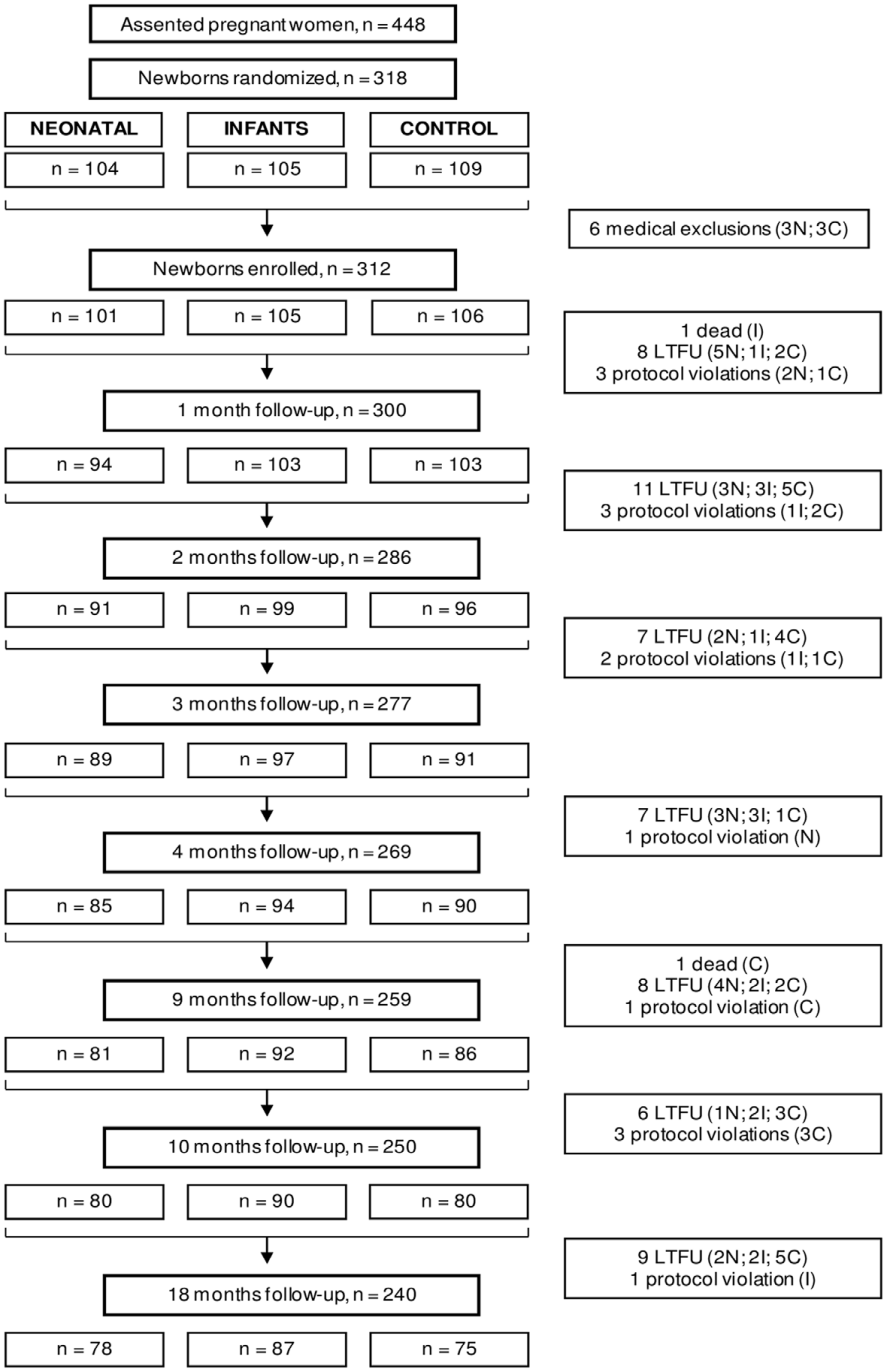
Flow diagram of the study [**31**]. Flow diagram indicating number of women who assented and children enrolled into the study, randomized to neonatal or infant PCV7 or control groups and number excluded or lost to follow-up in the course of the study. PCV = 7-valent pneumococcal conjugate vaccine; N = Neonatal group; I = Infant group; C = control group; LTFU = lost to follow-up (includes not located, withdrew consent, migration). Numbers (n) are total excluding LTFU and protocol violations.

Between May 3, 2005 and September 13, 2007, 318 newborns were randomized to the neonatal (104), infant (105) or control group (109) of which 101, 105 and 106 were subsequently enrolled in the respective groups. Figure 1 illustrates the number of children remaining in the study at different ages during the study period. Overall, 77% completed 18 months of follow-up (77%, 83% and 71% in the neonatal, infant and control groups, respectively, chi-squared = 4.36, 2 df, p = 0.11). Details of loss to follow-up and protocol violations have been reported previously [31]. Six children were excluded from analysis after a delayed diagnosis of contraindicated medical conditions, including HIV and congenital heart disease. There were two deaths during the study period, one from severe burns and gastroenteritis while the other died from pneumonia prior to receiving the scheduled PCV7; the post-PCV7 deaths were not related to the study vaccine [31]. Parental withdrawal and migration out of the study area were the main reasons for losses to follow-up in the first and later months of the trial, respectively. There were 14 protocol violations relating to allocated immunization schedule: 6 related to 7vPCV, 4 related to PPV and 4 children received DTP (at a government clinic) rather than DTP/Hib (Figure 1) [31].

The obstetric and newborn characteristics were similar in the neonatal, infant and control groups except for fewer boys in the neonatal group (43%) than in either infant (57%, p = 0.045) or control groups (61%, p = 0.008) (Table 1). No differences in population characteristics were found between children lost to follow-up and those followed to age 18 months (data not shown). Children received vaccines in a timely fashion at comparable ages [31].

**Table 1:** Obstetric and newborn characteristics according to randomization to neonatal or infant PCV7 schedule or to control group.

	Neonatal	Infant	Control
Age mother, years	25.7 (5.6)	25.3 (5.6)	25.4 (5.6)
Gravida:			
1	27%	36%	35%
2	30%	19%	24%
3	22%	21%	17%
4 or more	21%	24%	24%
Smoking in pregnancy	9%	4%	11%
Tetanus vaccination in pregnancy	94%	94%	97%
Maternal haemoglobin	12.2 (1.7)	12.1 (1.6)	12.1 (1.4)
Foetal distress	4%	1%	5%
Male newborn	43%	57%	61%
Delivery by caesarean	2%	2%	1%
Gestational age, weeks	39.4 (1.2)	39.5 (1.3)	39.6 (1.0)
Birth weight, g	3245 (440)	3310 (435)	3315 (520)
Birth length, cm	49.9 (3.0)	49.9 (3.9)	50.7 (3.3)
Head circumference, cm	33.2 (1.6)	33.5 (1.8)	33.5 (1.6)

Continuous variables are expressed as mean with standard deviation. Missing data resulted in varying denominators for the following variables: age mother, neonatal, n = 100; infant, n = 103; control, n = 108; smoking pregnancy, neonatal, n = 95; infant, n = 93; control, n = 102; tetanus vaccination, neonatal, n = 89; infant, n = 87; control, n = 94; foetal distress, neonatal, n = 89; infant, n = 85; control, n = 94; delivery, neonatal, n = 100; infant, n = 98; control, n = 107; gestational age, neonatal, n = 103; infant, n = 101; control, n = 102; birth weight, neonatal, n = 103; infant, n = 101; control, n = 102; birth length, neonatal, n = 98; infant, n = 97; control, n = 98; head circumference, neonatal, n = 102; infant, n = 94; control, n = 97; maternal haemoglobin, neonatal, n = 91; infant, n = 87; control, n = 83.

### Reactogenicity and Safety

No serious reactions were observed within an hour of vaccination. The vaccines were generally well tolerated (Table S1). Overall, children immunized according to the infant PCV7 schedule experienced more local side effects in response to PCV7 (22/287) than children vaccinated according to the neonatal PCV7 schedule (7/280, p = 0.020) and similar trends were observed for DTwP/Hib (infant group 48/284; neonatal group 26/261, p = 0.058) and Hepatitis B (infant group 8/288; neonatal groups 1/260, p = 0.059) (Table S1).

**Figure 2:**
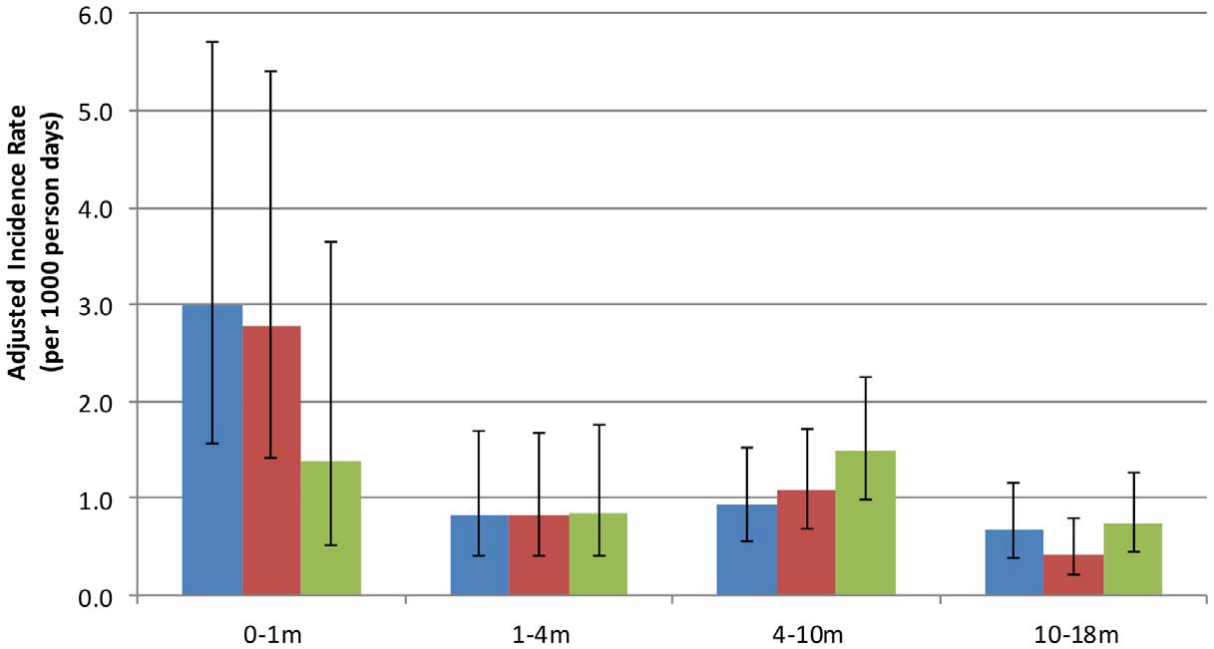
Risk for hospitalization. Gender-adjusted incidence rates of hospitalization (±95% confidence intervals) during the 1^st^ month, 1–<4 months (PCV immunization ongoing), 4–<10 months (PCV immunization completed), and 10–18 months (post-PPV) of life for the neonatal PCV (green bars), infant PCV (red bars) and control group (blue bars).

A total of 1243 illness episodes (355 in neonatal, 444 in infant and 444 in control groups) were documented from time of enrolment to completion of follow-up. Table S2 shows age-specific gender-adjusted incidence rates for moderate/severe pneumonia, all acute lower respiratory infections (ALRIs) and hospitalization rates in neonatal, infant and control groups. Adjusted incidence rates of acute lower respiratory infections (ALRIs) up to age 18 months were 1031 (n = 128), 1141 (n = 166) and 1275 (n = 162) per 1000 person-years at risk in the neonatal, infant and control groups, respectively, of which 40% were classified as moderate or severe pneumonia. There were no significant differences in ALRI incidence rates or hospitalization rates between neonatal, infant and control groups (Figure 2, Table S2). The number of SAEs was similar irrespective of vaccination schedule: 48 SAEs in the neonatal PCV7 group, 45 in the infant PCV7 group and 47 in children who had no PCV7 (379, 324 and 362/1000 person-years at risk, respectively). No SAEs were related to vaccination.

A total of 185 blood cultures were collected, 147 of which were from children aged <12 months. *Streptococcus pneumoniae* was isolated from 7 samples (3.8%, 95% CI 1.5–7.6%). All 7 isolates were from children aged <12 months, giving an estimated IPD incidence rate of 4762/100,000/annum (95% CI 1936–9665) in the first year of life. Three were isolated from children in the neonatal group (serotypes 5 and 8; the other was 12F isolated together with *H. influenzae* type C from an HIV–positive child subsequently excluded from the study). Serotype 19A was isolated from a child in the infant group and serotypes 2, 18C and serogroup 33 were isolated from unvaccinated children. No other significant pathogens were grown.

### Overview of Pneumococcal Serotype-Specific Antibody Responses Throughout the Study

**Figure 3:**
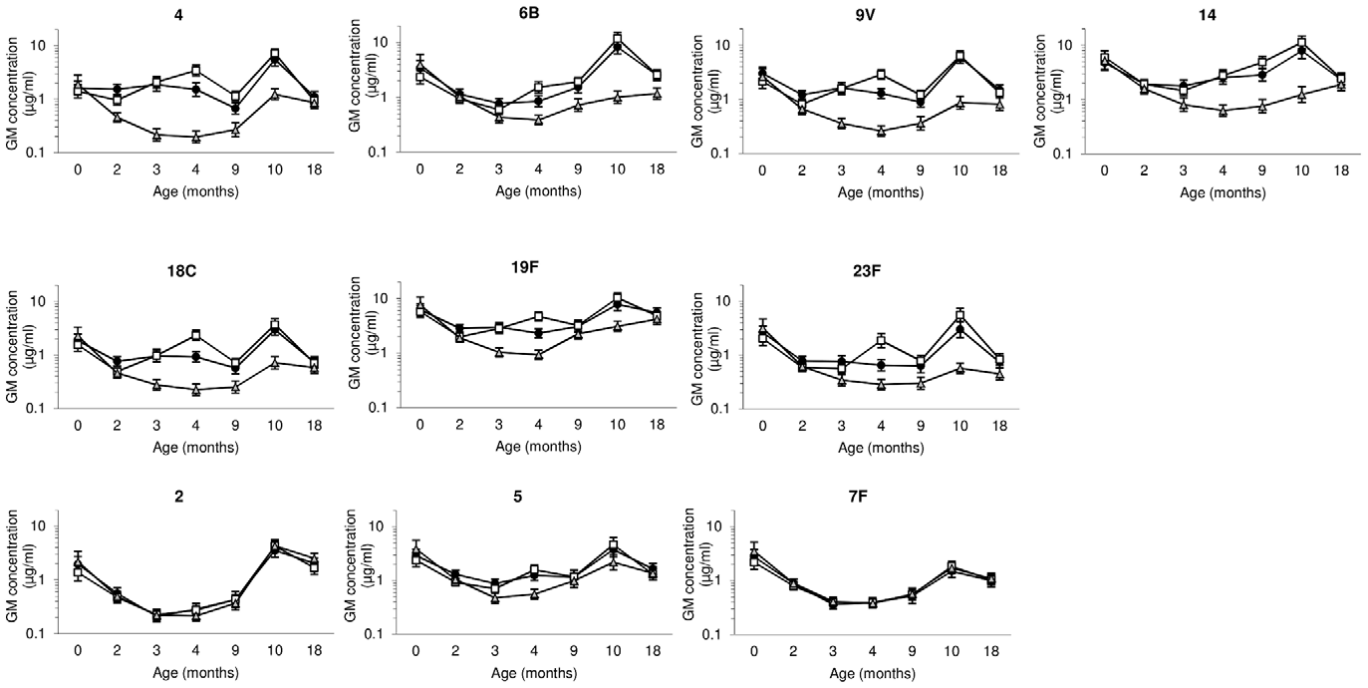
Age-specific geometric mean serotype-specific antibody concentrations pre-and post-PCV7 at 0-1-2 or 1-2-3-months or no PCV7. Geometric mean (GM) concentrations and 95% confidence intervals of serotype-specific IgG antibodies for PCV7 serotypes and non-PCV7 serotypes 2, 5 and 7F in the neonatal (•) (PCV7 at birth, 1 and 2 months), infant (□) (PCV7 at 1, 2, and 3 months) and control group (▵) (no PCV7). All children received PPV at 9 months of age.

Geometric mean antibody concentrations (and 95% CIs) for PCV7 serotypes and three NVTs from birth to age 18 months according to study group are shown in Figure 3. Table 2 shows the number of samples tested and the proportions of children with serotype-specific IgG concentration ≥0.35 µg/ml at each time point (with 95% CIs; shown graphically in Figure S1). GMCs and the proportions of samples with the more conservative cut-off of ≥1 µg/ml are in Table S3.

**Table 2:** Number of samples tested and percentage with serotype-specific antibody titre ≥0.35 µg/ml (95% CI) by age in neonatal and infant PCV7 groups and controls.

Age (mths)	Vaccine group	N^a^	PCV Serotypes, % (95% CI)							Non-PCV Serotypes, % (95% CI)		
			4	6B	9V	14	18C	19F	23F	2	5^b^	7F
Birth	Neonatal	44	93.2 (81.3–98.6)	95.5 (84.5–99.4)	97.7 (88.0–99.9)	100 (92.0–100)	95.5 (84.5–99.4)	100 (92.0–100)	95.5 (84.5–99.4)	90.9 (78.3–97.5)	100 (91.6–100)	97.7 (88.0–99.9)
	Infant	41	90.2 (76.9–97.3)	97.6 (87.1–99.9)	95.1 (83.5–99.4)	100 (91.4–100)	92.7 (80.1–98.5)	100 (91.4–100)	92.7 (80.1–98.5)	90.2 (76.9–97.3)	100 (91.2–100)	97.6 (87.1–99.9)
	Control	35	91.4 (76.9–98.2)	100 (90.0–100)	97.1 (85.1–99.9)	100 (90.0–100)	97.1 (85.1–99.9)	100 (90.0–100)	100 (90.0–100)	88.6 (73.3–96.8)	100 (89.7–100)	100 (90.0–100)
2	Neonatal	89	91.0 (83.1–96.0)	87.6 (79.0–93.7)	92.1 (84.5–96.8)	98.9 (93.9–100)	80.9 (71.2–88.5)	100 (95.9–100)	80.9 (71.2–88.5)	64.0 (53.2–73.9)	92.9 (85.3–97.4)	84.3 (75.0–91.1)
	Infant	93	86.0 (77.3–92.3)	85.0 (76.0–91.5)	83.9 (74.8–90.7)	95.7 (89.4–98.8)	71.0 (60.6–79.9)	97.8 (92.4–99.7)	73.1 (62.9–81.8)	60.2 (49.5–70.2)	87.4 (78.5–93.5)	82.8 (73.6–89.8)
	Control	95	67.4 (57.0–76.6)	87.4 (79.0–93.3)	77.9 (68.2–85.8)	93.7 (86.8–97.6)	57.9 (47.3–68.0)	97.9 (92.6–99.7)	75.8 (65.9–84.0)	61.1 (50.5–70.9)	88.8 (80.3–94.5)	87.4 (79.0–93.3)
3	Neonatal	85	90.6 (82.3–95.8)	78.8 (68.6–86.9)	92.9 (85.3–97.4)	91.8 (83.8–96.6)	84.7 (75.3–91.6)	98.8 (93.6–100)	74.1 (63.5–83.0)	27.1 (18.0–37.8)	86.3 (76.7–92.9)	56.5 (45.3–67.2)
	Infant	89	94.4 (87.4–98.2)	71.9 (61.4–80.9)	91.0 (83.1–96.0)	94.4 (87.4–98.2)	79.8 (69.9–87.6)	100 (95.9–100)	67.4 (56.7–77.0)	31.5 (22.0–42.2)	77.4 (67.0–85.8)	53.9 (43.0–64.6)
	Control	85	31.8 (22.1–42.8)	57.7 (46.4–68.3)	45.9 (35.0–57.0)	74.1(63.5–83.0)	41.2 (30.6–52.4)	88.2 (79.4–94.2)	54.1 (43.0–65.0)	29.4 (20.0–40.3)	59.2 (47.3–70.4)	57.6 (46.4–68.3)
4	Neonatal	85	90.6 (82.3–95.8)	80.0 (69.9–87.9)	92.9 (85.3–97.4)	94.1 (86.8–98.1)	85.9 (76.6–92.5)	98.8 (93.6–100)	71.8 (61.0–81.0)	40.0 (29.5–51.2)	90.5 (82.1–95.8)	57.6 (46.4–68.3)
	Infant	90	94.4 (87.5–98.2)	90.0 (81.9–95.3)	97.8 (92.2–99.7)	95.6 (89.0–98.8)	95.6 (89.0–98.8)	100 (96.0–100)	88.9 (80.5–94.5)	40.0 (29.8–50.9)	93.8 (86.2–98.0)	57.8 (46.9–68.1)
	Control	87	30.2 (20.8–41.1)	58.1 (47.0–68.7)	36.8 (26.7–47.8)	72.4 (61.8–81.5)	33.3 (23.6–44.3)	90.8 (82.7–95.9)	32.2 (22.6–43.1)	31.0 (21.5–41.9)	64.6 (53.3–74.9)	56.3 (45.3–66.9)
9	Neonatal	79	72.2 (60.9–81.7)	92.4 (84.2–97.2)	84.8 (75.0–91.9)	93.7 (85.8–97.9)	63.3 (51.7–73.9)	96.2 (89.3–99.2)	70.9 (59.6–80.6)	55.7 (44.1–66.9)	82.1 (69.6–91.1)	62.0 (50.4–72.7)
	Infant	87	90.8 (82.7–95.9)	98.9 (93.8–100)	90.8 (82.7–95.9)	95.4 (88.6–98.7)	77.0 (66.8–85.4)	98.9 (93.8–100)	77.0 (66.8–85.4)	63.2 (52.2–73.3)	91.1 (80.4–97.0)	63.2 (52.2–73.3)
	Control	83	43.4 (32.5–54.7)	75.6 (64.9–84.4)	50.6 (39.4–61.8)	68.7 (57.6–78.4)	42.2 (31.4–53.5)	96.4 (89.8–99.2)	47.0 (35.9–58.3)	56.6 (45.3–67.5)	81.0 (68.6–90.1)	71.1 (60.1–80.5)
10	Neonatal	77	96.1 (89.0–99.2)	98.7 (93.0–100)	100 (95.3–100)	94.8 (87.2–98.6)	98.7 (93.0–100)	100 (95.3–100)	90.9 (82.2–96.3)	94.8 (87.2–98.6)	98.2 (90.6–100)	88.3 (79.0–94.5)
	Infant	83	100 (95.7–100)	100 (95.7–100)	100 (95.7–100)	97.6 (91.6–99.7)	98.8 (93.5–100)	100 (95.7–100)	94.0 (86.5–98.0)	97.6 (91.6–99.7)	100 (93.7–100)	96.4 (89.8–99.2)
	Control	80	88.8 (79.7–94.7)	81.3 (71.0–89.1)	72.5 (61.4–81.9)	86.3 (76.7–92.9)	72.2 (60.9–81.7)	100 (95.5–100)	67.1 (55.6–77.3)	98.8 (93.2–100)	95.9 (86.0–99.5)	90.0 (81.2–95.6)
18	Neonatal	79	85.0 (75.3–92.0)	96.3 (89.4–99.2)	92.4 (84.2–97.2)	94.9 (87.5–98.6)	76.0 (65.0–84.9)	100 (95.4–100)	75.9 (65.0–84.9)	95.0 (87.7–98.6)	96.6 (88.3–99.6)	78.5 (67.8–86.9)
	Infant	86	81.4 (71.6–89.0)	98.8 (93.7–100)	95.4 (88.5–98.7)	95.4 (88.5–98.7)	70.9 (60.1–80.2)	100 (95.8–100)	80.2 (70.2–88.0)	93.0 (85.4–97.4)	93.3 (83.8–98.2)	82.6 (72.9–89.9)
	Control	76	79.0 (68.1–87.5)	86.8 (77.1–93.5)	76.3 (65.2–85.3)	92.1 (83.6–97.0)	65.8 (54.0–76.3)	100 (95.3–100)	60.5 (48.6–71.6)	97.4 (90.8–99.7)	95.7 (85.2–99.5)	86.8 (77.1–93.5)

a Number of assays performed.

b Number of assays performed for serotype 5: birth 34–42, 2 months 85–89, 3 months 76–84, 4 months 81–84, 9 months 56–58, 10 months 49–57, 18 months 46–60.

Fewer samples were available for pneumococcal antibody assays at birth than at subsequent time points as study personnel were not always available at the time of delivery (Table 2). Generally, serum volumes were adequate to measure antibodies to all serotypes. Fewer assays were conducted for serotype 5 in view of some concerns regarding the reliability of the assay.

The high levels of maternal antibody in all groups at birth declined over the first 2 months of life; in the control group the decline continued to its lowest levels at age 4 months (Figure 3). VT antibody concentrations were higher in both infant and neonatal PCV7 groups than in controls from 2 to 9 months of age for serotype 4 and from 3 to 9 months of age for all other PCV7 serotypes except for serotype 19F for which GMCs were high in all groups, with no difference between vaccine and control groups at age 9 months (Figure 3, Table S3). From 2 to 10 months of age more children (generally >80%) in both PCV groups had antibody concentrations ≥0.35 µg/ml for all VTs than in the control group, except for serotype 19F, where the proportion with a protective level in the control group were also high (Figure S1). The more conservative 1 µg/ml cut-off was more discriminatory in showing differences in antibody responses between vaccinated and unvaccinated children (Table S3). At age 4 months GMCs of VT-specific antibody were higher in the infant group (one month after the third dose of PCV7) than in the neonatal group that had received the third dose of PCV7 two months earlier (Figure 3, Table S3); a comparable high proportion of children in both groups had antibody concentrations ≥0.35 µg/ml for PCV7 serotypes (averages varying between 72 and 98%), except for serotype 23F for which fewer children in the neonatal than infant group had concentrations ≥0.35 µg/ml (Figure S1). Generally high antibody concentrations persisted to 18 months of age with >75% of children having antibody concentrations ≥0.35 µg/ml for PCV7 serotypes (and >90% for PCV7 serotypes 6B, 9V, 14 and 19F).

IgG concentrations to non-PCV7 serotypes 2 and 7F were similar in PCV7-vaccinated and control groups at all ages, the lowest levels being at age 3–4 months. For non-PCV7 serotype 5, IgG concentrations and the proportion of samples with concentration ≥0.35 µg/ml were higher at ages 3–4 months in children given PCV7 than in controls (Figure 3, Table 2, Table S3, Figure S1). Responses for NVTs one month after PPV at age 9 months were good in all children (mean fold increases 2.9–3.3 for serotype 7F, 2.2–3.3 for serotype 5 and 8–11.6 for serotype 2) and antibody levels were maintained to age 18 months.

There were no significant differences in serotype-specific IgG concentrations between males and females.

### Immunogenicity of PCV Under the Standard PNG 1-2-3-month Schedule

At age 2 months IgG concentrations for serotypes 4 and 9V were significantly higher one month after the first dose of PCV7 than in controls (serotype 4 GMC 0.96 µg/ml (95%CI 0.78–1.18) in infant group vs 0.46 (0.37–0.56) in control group, p<0.001; serotype 9V 0.82 µg/ml (0.69–0.96) in infant group vs 0.66 µg/ml (0.53–0.84) in controls, p = 0.044); there were no significant differences for the other VTs at this age. However, GMCs for VTs, other than serotype 6B, were significantly higher in the vaccinated (GMC range 0.56–2.82 µg/ml) than in the unvaccinated group (GMC range 0.21–1.03 µg/ml) one month after the 2^nd^ dose (age 3 months) and for all VTs after the 3^rd^ dose (age 4 months) (GMC 1.52–4.68 vs 0.2–0.94 µg/ml) (Figure 3 and Table S3). There were no statistical differences in the proportions of children with antibody concentration ≥0.35 µg/ml at 2 months of age between the infant group (range 71.0–97.8%) and controls (57.9–97.9%); however, at 3 months of age this proportion was significantly higher in the infant group than in unvaccinated children for 5 of the 7 VT serotypes (serotype 4 (94.4% vs 31.8%), 9V (91.0% vs 45.9%), 14 (94.4% vs 74.1%), 18C (79.8% vs 41.2%) and 19F (100% vs 88.2%)) and at 4 months of age for all 7 VT serotypes (range 90.0–100% vs 30.2–90.8%) (Table 2 and Figure S1).

### Immunogenicity of Neonatal Immunization with PCV

Despite the high background levels of maternally derived antibody (generally >80% had levels ≥0.35 µg/ml), GMCs were significantly higher at 2 months of age in the neonatal than in the infant group for 4 of the 7 VTs (serotypes 4 (GMC 1.54 µg/ml (95%CI 1.24–1.91) vs 0.96 (0.78–1.18), p = 0.002), 9V (1.21 (1.02–1.45) vs 0.82 (0.69–0.96), p = 0.008), 18C (0.76 (0.62–0.93) vs 0.49 (0.42–0.57), p = 0.001) and 19F (2.84 (2.42–3.34) vs 1.98 (1.69–2.32), p = 0.003), Figure 3 and Table S3). The proportions with antibody level ≥0.35 µg/ml were higher in the neonatal than in the infant group for all VTs at 2 months of age (range 80.9–100% vs 71–97.8%), but differences between the groups were not significant as the majority of children had antibody levels above this cut-off (Table 2). Post-hoc analysis of the differences in proportions of children with protective antibody levels at age 2 months did not demonstrate superiority of 2 doses in the neonatal group over 1 dose in the infant group (Figure S2). However, all antibody responses to VTs in the neonatal group were non-inferior to those in the infant group at age 2 months (Figure S2).

### Induction of Immunologic Memory Following Neonatal and Early Infant PCV7 Schedules

**Figure 4:**
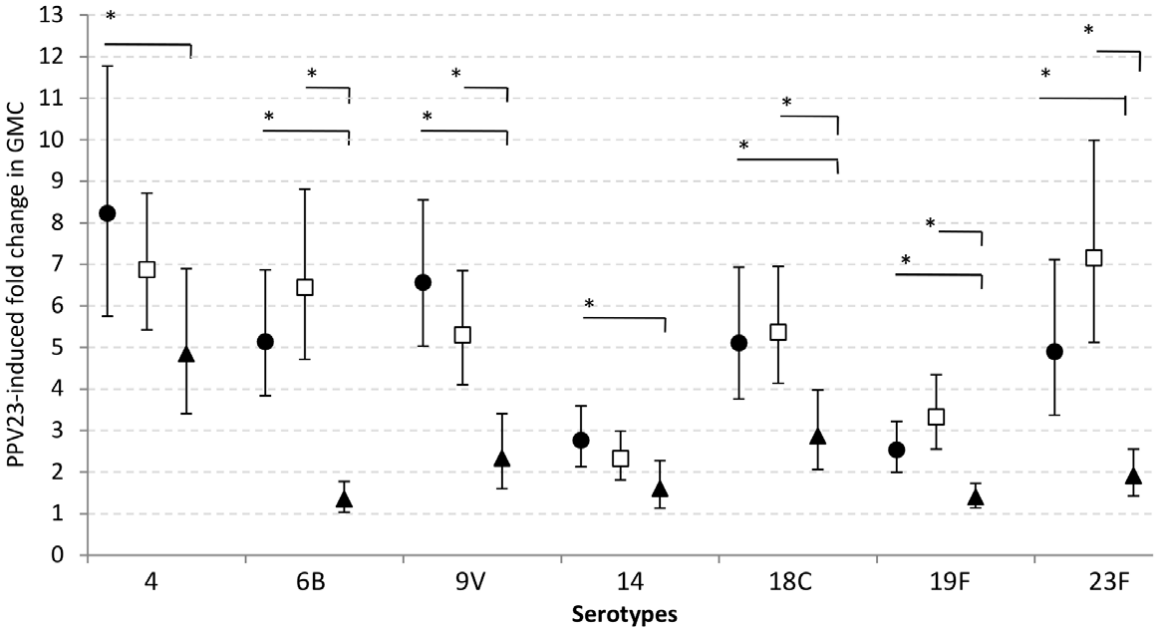
Geometric mean fold rise in antibody for PCV7-serotypes following PPV at age 9 months. To demonstrate immunological memory, PPV was administered at 9 months of age and the mean (and 95% CI) fold changes in PCV7-serotype-specific GMCs were calculated and compared for the neonatal (•), infant (□) and control group (▴), with significant differences between groups (p<0.05) indicated with *.

PPV given at 9 months of age induced antibody responses in both PCV7-primed and unprimed infants, as demonstrated by the rise in serotype-specific antibody concentrations between 9 and 10 months of age (Figure 3 and Table S3). However, average fold change in serotype-specific IgG concentrations between pre- and post-PPV vaccination (Figure 4) was greater in PCV7-primed than in unprimed children (except for serotypes 4 and 14 in the infant group for which the fold change was not significantly different from that in the control group). The mean increase in the VT IgG concentration was 6.4-fold in the PCV-vaccinated groups compared with 1.9-fold in the control group. There were no significant differences in the PPV-induced rise in IgG concentrations between the neonatal and infant vaccination groups. At age 10 months antibody levels were significantly higher in children primed with PCV7 than in the control group (GMC range 2.98–11.83 µg/ml vs 0.57–3.11) with no difference between the neonatal and infant groups except for serotype 23F, for which levels were higher in the infant (GMC 5.55 µg/ml (95%CI 4.15–7.41)) than in the neonatal group (GMC 3.00 µg/ml (2.13–4.24), p = 0.009) (Table S3). At age 10 months, one month post-PPV, 91–100% of PCV-primed children had antibody levels ≥0.35 µg/ml compared with 67–100% in children who had received only PPV, the lowest proportion being for serotype 23F (91% in neonatal, 94% in infant and 67% in control groups). All children had antibody levels ≥0.35 µg/ml to serotype 19F (Table 2).

## Discussion

This is one of only two studies of neonatal PCV and the only study to include an unimmunized control group and a booster with a standard dose of PPV. Follow-up to age 18 months and detailed investigation of T-cell responses have provided important safety data, especially immunological safety.

We found that PCV7 is well tolerated and immunogenic in the PNG standard 1-2-3-month schedule, relevant to PNG policy-makers who plan to introduce a universal PCV program in 2013. The neonatal PCV7 schedule was not only safe and immunogenic, but GMCs for 4 of the PCV7 serotypes were higher at age 2 months in the neonatal than in the standard schedule group, despite high levels of maternally derived antibody limiting the ability to measure infant-derived antibody responses. With high maternal antibody titres declining at different rates, the impact of a neonatal priming PCV dose is hard to measure as the response to the second dose of PCV is measured at 2 months of age when a significant proportion of maternal antibody is still present. We cannot exclude that neonatal PCV responses are limited by interference of maternal antibodies or a reduced capacity of neonatal B cells to respond to some serotypes in PCV7. We did not find impairment in the overall response to the 3 dose PCV series when started at birth. Assessment of polysaccharide-specific memory B-cells or functional opsonophagocytic antibody may inform this issue. Importantly, we have previously reported that neonatal PCV7 primes T-cell responses with no bystander effects on other T-cell responses and no evidence of T-cell tolerance [38], [39]. There was also no clinical evidence of deleterious effects of neonatal immunization: in fact neonatal immunization appeared to reduce the risk of local reactions to PCV7 and possibly to other infant vaccines. The reduction in local reactogenicity after neonatal immunization is an interesting and potentially important finding but to establish the reason for this would require further specific investigation. It should be noted that, after an initial 1-hour post-vaccination examination, for logistical reasons we assessed reactogenicity 48–96 hours post-vaccination. If we had been able to assess children earlier than 48 hours post-vaccination, we might have detected more reactions to vaccination, though, importantly, no serious side effects following neonatal or early infant PCV7 schedules were identified. While we have presented ALRI incidence data, the study was not powered to formally investigate the impact of accelerated PCV schedules on ALRI morbidity.

The high levels of maternal antibody in early infancy and the differences at specific time points between the neonatal and infant groups with regard to the number of doses and the time elapsed since last dose have to be considered when comparing antibody responses between neonatal and infant schedules: for example, at age 2 months the neonatal group will have received 2 doses compared with only 1 in the infant group, while at age 4 months, 2 months will have elapsed since the 3^rd^ dose of PCV7 in the neonatal group but only 1 month since the last dose in the infant PCV7 group. The latter may explain lower serotype-specific antibody levels in the neonatal than in the infant group at age 4 months. This difference was no longer evident at age 9 months.

Both infant and neonatal PCV7 schedules induced immunological memory to a PPV booster at age 9 months with higher VT antibody levels one month post-PPV than in unprimed children that were generally maintained to age 18 months. Children also elicited good IgG responses to non-PCV7 serotypes 2, 5 and 7 following PPV immunization. However, we discontinued measurement of IgG to non-PCV7 serotype 5 in view of the higher titres for this serotype seen in PCV7-immunized children than in controls. Kieninger *et al* also found antibody responses to serotype 5 following PCV7 but no functional activity, suggesting cross-reactivity with another bacterial polysaccharide which requires further investigation [40]. Functional antibody assays could not be done on samples from our PNG study until now due to financial constraints but measurement of opsonophagocytic antibody assays to all PCV7 serotypes will commence shortly.

We included a standard PPV dose at age 9 months not only to investigate immunological memory but also because of the demonstrated efficacy of PPV in prevention of death and serious ALRI in an earlier study among Papua New Guinean children [23], [25]. A PPV booster following PCV priming could provide the broader serotype coverage required in high-risk populations and assist in preventing replacement disease reported following introduction of PCVs [41], [42], [43]. The good antibody responses to PPV at age 9 months in PCV7-primed and unprimed Papua New Guinean children were consistent with our earlier study in PNG [24] and with those in Fiji, Kenya and The Gambia [22], [44], [45], [46]. However, there have been concerns of hyporesponsiveness following PPV vaccination [47], [48]. While the clinical significance of hyporesponsiveness remains unclear, this phenomenon requires further investigation, specifically in populations with high pneumococcal carriage rates. Preliminary data following a challenge dose of PPV at age 3–5 years in children who took part in the present PNG study compared to unimmunized controls showed no deleterious effect on antibody persistence or responses to a challenge dose and no difference in pneumococcal carriage rates between groups (ClinicalTrials.gov NCT01414504) [49], [50]. However, there was no group of children who received PCV with no PPV and there was a prolonged time interval between PPV and challenge. Therefore another study is underway in PNG in which we are investigating immunological memory at age 23 months following PPV at age 9 months in children primed either with 10-valent or 13-valent PCV (ClinicalTrials.gov NCT01619462).

Our findings are consistent with those from the only other study of neonatal PCV7, conducted in Kenya [22], in which antibody responses were similar following PCV7 at 0-6-14 weeks or 6-10-14 weeks. Antibody levels in the Kenyan study were remarkably similar at age 5 months to our findings at age 4 months (one month after 3 doses in Kenya compared with 2 months after 3 doses of PCV7 in PNG). However, more children had serotype-specific antibody titres ≥1 µg/ml at 18 weeks following the 0-10-14-week PCV7 schedule in Kenya than at 16 weeks in PNG following a 0-4-8-week schedule, suggesting that a longer interval between neonatal and second dose may be preferable.

Interestingly, a high proportion of children in our study, including those in the control group, had antibody concentrations ≥0.35 µg/ml to serotype 19F throughout the study period. This serotype was the most commonly carried in the population.

The United Nations’ 4^th^ Millennium Development Goal (MDG) aims to reduce childhood mortality by two-thirds by 2015 and WHO has recommended investigation of neonatal immunization to reduce the high mortality in early infancy [51]. This MDG will not be achieved without addressing the enormous burden of pneumonia and neonatal mortality [52]. With pneumonia responsible for more than 2 million childhood deaths, the majority in the first 6 months of life, early immunization is vital. The inclusion of a neonatal dose of PCV would achieve this and also improve PCV coverage [53], [54] without compromising effectiveness. The additional use of a PPV booster may also offer protection against important non-PCV serotypes such as serotype 2 [14], [28] as well as boosting for the PCV serotypes. These results justify further studies of neonatal immunization, including comprehensive clinical trials with second generation PCVs covering additional important serotypes causing invasive pneumococcal disease.

## Supporting Information

Figure S1 **Proportions of children with serotype-specific antibody titre** ≥**0.35 µg/ml by age in PCV7-immunized and unimmunized children.** The proportions of children (and 95% confidence intervals) with IgG antibody concentration above the serologic correlate of protection (≥0.35 µg/ml), specific for PCV7 serotypes and non-PCV7 serotypes (2, 5 and 7F), in the neonatal (•) (PCV7 at birth, 1 and 2 months), infant (□) (PCV7 at 1, 2, and 3 months) and control group (▴) (no PCV7). All children received PPV at 9 months of age.
(TIF)

Figure S2 **Superiority or non-inferiority of the neonatal versus infant PCV7 schedule at 2 months of age.** We calculated the exact, unconditional, 2-sided 95% CIs on the difference in proportions of children with serotype-specific IgG concentration ≥0.35 µg/ml after 2 doses of PCV7 in the neonatal group (at birth and 1 month) compared with that found after 1 dose of PCV7 in the infant group (at 1 month of age). The results indicate that the responses in the neonatal group were non-inferior to those in the infant group.
(TIF)

Table S1 **Frequency of local and systemic side effects to PCV7, PPV and routine EPI vaccines.**
(DOCX)

Table S2 **Age-specific incidence rates (/1000 person-years), number of cases (n) and upper and lower 95% confidence limits for rates (95% CL, LCL and UCL) of (A) moderate/severe pneumonia, (B) any acute lower respiratory infection (ALRI) and (C) any hospitalization among children who received 7-valent pneumococcal conjugate vaccine in a 0-1-2-month (neonatal group) or a 1-2-3-month (infant group) schedule and among controls.**
(DOCX)

Table S3 **Geometric mean antibody concentrations (GMC) and 95% confidence intervals (95%CI) and percentage with serotype-specific antibody titre ≥1 µg/ml (95% CI) by age in neonatal and infant PCV7 groups and controls.**
(DOCX)

File S1 **List of institutions and investigators comprising the Neonatal Pneumococcal Conjugate Vaccine Trial Study Team.**
(DOCX)

Protocol S1 **Trial Protocol.**
(DOC)

Checklist S1 **CONSORT checklist.**
(DOC)
