# Supplementary material for: Safety and Immunogenicity of Neonatal Pneumococcal Conjugate Vaccination in Papua New Guinean Children: A Randomised Controlled Trial
Source: PLoS One. 2013 Feb 22;8(2):e56698. doi: 10.1371/journal.pone.0056698 (PMC3579820; doi:10.1371/journal.pone.0056698)
Supplement: Table S1 — Frequency of local and systemic side effects to PCV7, PPV and routine EPI vaccines. (DOCX) [file pone.0056698.s003.docx]

**Table S1. Frequency of local and systemic side effects to PCV7, PPV and routine EPI vaccines.**

|  | **Birth** | | | **1 month** | | | **2 months** | | | **3 months** | | | **9 months** | | | **Total** | | | |
| --- | --- | --- | --- | --- | --- | --- | --- | --- | --- | --- | --- | --- | --- | --- | --- | --- | --- | --- | --- |
|  | **N** | **I** | **C** | **N** | **I** | **C** | **N** | **I** | **C** | **N** | **I** | **C** | **N** | **I** | **C** | **N** | **I** | **C** | **P-Value** |
| **PCV7** |  |  |  |  |  |  |  |  |  |  |  |  |  |  |  |  |  |  |  |
| Redness | 0 | - | - | 0 | 0 | - | 2 | 4 | - | - | 1 | - | - | - | - | 2 (1%) | 5 (2%) | - | 0.278 |
| Swelling | 1 | - | - | 0 | 2 | - | 0 | 1 | - | - | 0 | - | - | - | - | 1 (<1%) | 3 (1%) | - | 0.349 |
| Tenderness | 0 | - | - | 2 | 4 | - | 4 | 11 | - | - | 5 | - | - | - | - | 6 (2%) | 20 (7%) | - | 0.028 |
| **PPV** |  |  |  |  |  |  |  |  |  |  |  |  |  |  |  |  |  |  |  |
| Redness | - | - | - | - | - | - | - | - | - | - | - | - | 0 | 0 | 0 | 0 (0%) | 0 (0%) | 0 (0%) | N/A |
| Swelling | - | - | - | - | - | - | - | - | - | - | - | - | 0 | 0 | 0 | 0 (0%) | 0 (0%) | 0 (0%) | N/A |
| Tenderness | - | - | - | - | - | - | - | - | - | - | - | - | 2 | 0 | 0 | 2 (3%) | 0 (0%) | 0 (0%) | 0.101 |
| **BCG** |  |  |  |  |  |  |  |  |  |  |  |  |  |  |  |  |  |  |  |
| Redness | 0 | 1 | 0 | - | - | - | - | - | - | - | - | - | - | - | - | 0 (0%) | 1 (1%) | 0 (0%) | 0.408 |
| Swelling | 0 | 1 | 1 | - | - | - | - | - | - | - | - | - | - | - | - | 0 (0%) | 1 (1%) | 1 (1%) | 0.667 |
| Tenderness | 1 | 1 | 2 | - | - | - | - | - | - | - | - | - | - | - | - | 1 (1%) | 1 (1%) | 2 (2%) | 0.821 |
| **DTwP/Hib** |  |  |  |  |  |  |  |  |  |  |  |  |  |  |  |  |  |  |  |
| Redness | - | - | - | 2 | 3 | 5 | 9 | 5 | 8 | 2 | 5 | 7 | - | - | - | 13 (5%) | 13 (5%) | 20 (8%) | 0.319 |
| Swelling | - | - | - | 2 | 7 | 4 | 1 | 3 | 3 | 1 | 1 | 3 | - | - | - | 4 (2%) | 11 (4%) | 10 (4%) | 0.230 |
| Tenderness | - | - | - | 7 | 11 | 17 | 10 | 19 | 9 | 7 | 13 | 10 | - | - | - | 24 (9%) | 43 (15%) | 36 (14%) | 0.231 |
| **Hepatitis B** |  |  |  |  |  |  |  |  |  |  |  |  |  |  |  |  |  |  |  |
| Redness | 0 | 0 | 1 | 0 | 0 | 0 | - | - | - | 0 | 1 | 0 | - | - | - | 0 (0%) | 1 (<1%) | 1 (<1%) | N/A |
| Swelling | 0 | 1 | 0 | 0 | 2 | 2 | - | - | - | 0 | 1 | 0 | - | - | - | 0 (0%) | 4 (1%) | 2 (<1%) | 0.164 |
| Tenderness | 0 | 0 | 0 | 0 | 1 | 3 | - | - | - | 1 | 3 | 1 | - | - | - | 1 (<1%) | 4 (1%) | 4 (1%) | 0.452 |
| **Measles** |  |  |  |  |  |  |  |  |  |  |  |  |  |  |  |  |  |  |  |
| Redness | - | - | - | - | - | - | - | - | - | - | - | - | 0 | 1 | 0 | 0 (0%) | 1(1%) | 0 (0%) | 0.441 |
| Swelling | - | - | - | - | - | - | - | - | - | - | - | - | 0 | 1 | 0 | 0 (0%) | 1(1%) | 0 (0%) | 0.441 |
| Tenderness | - | - | - | - | - | - | - | - | - | - | - | - | 0 | 1 | 0 | 0 (0%) | 1(1%) | 0 (0%) | 0.441 |
| **Systemic** |  |  |  |  |  |  |  |  |  |  |  |  |  |  |  |  |  |  |  |
| Vomiting | 0 | 0 | 1 | 0 | 0 | 0 | 0 | 0 | 0 | 0 | 0 | 0 | 1 | 0 | 1 | 1 (<1%) | 0 (0%) | 2 (<1%) | N/A |
| Diarrhoea | 0 | 1 | 0 | 0 | 1 | 0 | 0 | 1 | 2 | 0 | 0 | 0 | 0 | 2 | 2 | 0 (0%) | 5 (1%) | 4 (<1%) | N/A |
| Irritability | 4 | 0 | 2 | 11 | 7 | 12 | 8 | 8 | 6 | 5 | 4 | 7 | 2 | 2 | 2 | 30 (7%) | 21 (4%) | 29 (7%) | 0.366 |
| Drowsiness | 0 | 0 | 0 | 0 | 0 | 0 | 0 | 0 | 0 | 0 | 0 | 0 | 0 | 0 | 0 | 0 (0%) | 0 (0%) | 0 (0%) | N/A |
| Rash | 0 | 0 | 0 | 0 | 0 | 0 | 1 | 0 | 0 | 0 | 0 | 0 | 0 | 0 | 1 | 1 (<1%) | 0 (0%) | 1 (<1%) | N/A |

N = neonatal; I = infant; and C = control group. Reactogenicity to measles vaccination was only recorded for the 2^nd^ dose administered at 9 months and not after the 1^st^ dose given at 6 months as this was frequently given through the routine health services.

P value relates to differences between the 3 groups.

N/A = not applicable.
