# Supplementary material for: Safety and Immunogenicity of Neonatal Pneumococcal Conjugate Vaccination in Papua New Guinean Children: A Randomised Controlled Trial
Source: PLoS One. 2013 Feb 22;8(2):e56698. doi: 10.1371/journal.pone.0056698 (PMC3579820; doi:10.1371/journal.pone.0056698)
Supplement: Table S2 — Age-specific incidence rates (/1000 person-years), number of cases (n) and upper and lower 95% confidence limits for rates (95% CL, LCL and UCL) of (A) moderate/severe pneumonia, (B) any acute lower respiratory infection (ALRI) and (C) any hospitalization among children who received 7-valent pneumococcal conjugate vaccine in a 0-1-2-month (neonatal group) or a 1-2-3-month (infant group) schedule and among controls. (DOCX) [file pone.0056698.s004.docx]

**Table S2. Age-specific incidence rates (/1000 person-years), number of cases (n) and upper and lower 95% confidence limits for rates (95% CL, LCL and UCL) of (A) moderate/severe pneumonia, (B) any acute lower respiratory infection (ALRI) and (C) any hospitalization among children who received 7-valent pneumococcal conjugate vaccine in a 0-1-2-month (neonatal group) or a 1-2-3-month (infant group) schedule and among controls.**

|  | | |  | |  |  |  |  |  |  |  |  |  |  |
| --- | --- | --- | --- | --- | --- | --- | --- | --- | --- | --- | --- | --- | --- | --- |
|  | **Neonatal group** | | | |  | **Infant group** | | | |  | **Control group** | | | |
| Age in months | rate | n | 95% CL | | | rate | n | 95% CL | | | rate | n | 95% CL | |
|  |  |  | LCL | UCL |  |  |  | LCL | UCL |  |  |  | LCL | UCL |
| *(A) Moderate/severe pneumonia* |  |  |  |  |  |  |  |  |  |  |  |  |  |  |
| 0-<1 | 470.90 | 4 | 172.32 | 1287.35 |  | 383.27 | 4 | 133.67 | 1099.20 |  | 453.93 | 5 | 171.29 | 1202.72 |
| 1-<4 | 612.36 | 14 | 362.61 | 1034.15 |  | 595.76 | 16 | 359.72 | 986.67 |  | 637.04 | 17 | 388.13 | 1045.59 |
| 4-<10 | 712.25 | 30 | 497.87 | 1018.76 |  | 500.13 | 24 | 333.73 | 749.45 |  | 488.58 | 23 | 322.58 | 740.51 |
| 10-18 | 249.96 | 13 | 144.80 | 431.53 |  | 238.67 | 16 | 142.21 | 400.35 |  | 281.57 | 17 | 168.91 | 469.34 |
| 0-18 | 489.44 | 61 | 382.35 | 631.65 |  | 404.33 | 60 | 311.79 | 523.92 |  | 438.67 | 62 | 338.78 | 568.12 |
|  |  |  |  |  |  |  |  |  |  |  |  |  |  |  |
| *(B) Any ALRI* |  |  |  |  |  |  |  |  |  |  |  |  |  |  |
| 0-<1 | 740.73 | 6 | 331.40 | 1656.29 |  | 738.90 | 7 | 340.81 | 1602.53 |  | 708.22 | 7 | 323.22 | 1552.06 |
| 1-<4 | 1446.39 | 33 | 1027.97 | 2035.18 |  | 1446.39 | 38 | 1,043.50 | 1998.87 |  | 1728.36 | 45 | 1277.09 | 2341.01 |
| 4-<10 | 1253.54 | 53 | 957.51 | 1641.45 |  | 1325.86 | 63 | 1,032.09 | 1698.22 |  | 1375.17 | 64 | 1072.06 | 1764.00 |
| 10-18 | 699.45 | 36 | 504.38 | 970.04 |  | 902.17 | 58 | 688.38 | 1180.08 |  | 798.44 | 46 | 589.54 | 1081.75 |
| 0-18 | 1031.47 | 128 | 867.26 | 1227.01 |  | 1141.04 | 166 | 976.85 | 1333.20 |  | 1174.64 | 162 | 1002.59 | 1376.55 |
|  |  |  |  |  |  |  |  |  |  |  |  |  |  |  |
| *(C) Any hospitalization* | |  |  |  |  |  |  |  |  |  |  |  |  |  |
| 0-<1 | 501.47 | 4 | 188.17 | 1335.87 |  | 1013.30 | 9 | 519.23 | 1977.00 |  | 1093.24 | 10 | 573.83 | 2082.53 |
| 1-<4 | 306.41 | 7 | 145.96 | 643.12 |  | 299.43 | 8 | 146.84 | 610.54 |  | 301.62 | 8 | 146.84 | 619.76 |
| 4-<10 | 546.02 | 23 | 362.61 | 822.49 |  | 396.02 | 19 | 251.47 | 624.12 |  | 339.98 | 16 | 206.71 | 559.11 |
| 10-18 | 271.61 | 14 | 160.67 | 458.67 |  | 149.28 | 9 | 77.12 | 288.97 |  | 244.16 | 13 | 139.82 | 426.38 |
| 0-18 | 386.07 | 48 | 291.00 | 512.01 |  | 313.02 | 45 | 232.60 | 421.30 |  | 345.89 | 47 | 258.10 | 463.75 |
