# Supplementary material for: Safety and Immunogenicity of Neonatal Pneumococcal Conjugate Vaccination in Papua New Guinean Children: A Randomised Controlled Trial
Source: PLoS One. 2013 Feb 22;8(2):e56698. doi: 10.1371/journal.pone.0056698 (PMC3579820; doi:10.1371/journal.pone.0056698)
Supplement: Table S3 — Geometric mean antibody concentrations (GMC) and 95% confidence intervals (95%CI) and percentage with serotype-specific antibody titre ≥1 µg/ml (95% CI) by age in neonatal and infant PCV7 groups and controls. (DOCX) [file pone.0056698.s005.docx]

**Table S3. Geometric mean antibody concentrations (GMC) and 95% confidence intervals (95%CI) and percentage with serotype-specific antibody titre ≥1µg/ml (95% CI) by age in neonatal and infant PCV7 groups and controls.**

|  |  | **GMC µg/ml (95%CI)** | | | **Percentage ≥1µg/ml (95% CI)** | | |
| --- | --- | --- | --- | --- | --- | --- | --- |
| **Serotype** | **Age (months)** | **Neonatal** | **Infant** | **Control** | **Neonatal** | **Infant** | **Control** |
| **PCV7** |  |  |  |  |  |  |  |
| **4** | Birth | 1.62 (1.20-2.17) | 1.41 (1.06-1.87) | 1.89 (1.28-2.81) | 72.7 (57.2-85.0) | 70.7 (54.5-83.9) | 71.4 (53.7-85.4) |
|  | 2 mo | 1.54 (1.24-1.91) | 0.96 (0.78-1.18) | 0.46 (0.37-0.56) | 65.2 (54.3-75.0) | 47.3 (36.9-57.9) | 18.9 (11.6-28.3) |
|  | 3 mo | 1.87 (1.40-2.48) | 2.08 (1.63-2.66) | 0.21 (0.17-0.28) | 80.0 (69.9-87.9) | 75.3 (65.0-83.8) | 5.9 (1.9-13.2) |
|  | 4 mo | 1.52 (1.14-2.03) | 3.44 (2.72-4.35) | 0.20 (0.15-0.25) | 70.6 (59.7-80.0) | 87.8 (79.2-93.7) | 7.0 (2.6-14.6) |
|  | 9 mo | 0.67 (0.53-0.85) | 1.14 (0.93-1.40) | 0.27 (0.20-0.36) | 36.7 (26.1-48.3) | 57.5 (46.4-68.0) | 15.7 (8.6-25.3) |
|  | 10 mo | 5.51 (4.16-7.30) | 7.13 (5.91-8.61) | 1.24 (0.98-1.57) | 88.3 (79.0-94.5) | 98.8 (93.5-100) | 60.0 (48.4-70.8) |
|  | 18 mo | 1.11 (0.90-1.38) | 0.93 (0.75-1.16) | 0.86 (0.67-1.09) | 51.3 (39.8-62.6) | 51.2 (40.1-62.1) | 44.7 (33.3-56.6) |
| **6B** | Birth | 3.41 (2.47-3.92) | 2.35 (1.77-3.12) | 3.98 (2.61-6.08) | 88.6 (75.4-96.2) | 82.9 (67.9-92.9) | 88.6 (73.3-96.8) |
|  | 2 mo | 1.15 (0.93-1.41) | 0.93 (0.77-1.13) | 1.03 (0.85-1.24) | 57.3 (46.4-67.7) | 46.2 (35.8-56.9) | 49.5 (39.1-59.9) |
|  | 3 mo | 0.77 (0.63-0.96) | 0.59 (0.49-0.72) | 0.44 (0.34-0.56) | 40.0 (29.5-51.2) | 24.7 (16.2-35.0) | 24.7 (16.0-35.3) |
|  | 4 mo | 0.85 (0.67-1.08) | 1.52 (1.20-1.94) | 0.39 (0.32-0.47) | 43.5 (32.8-54.7) | 71.1 (60.6-80.2) | 11.6 (5.7-20.4) |
|  | 9 mo | 1.53 (1.19-1.97) | 1.92 (1.60-2.30) | 0.72 (0.55-0.94) | 65.8 (54.3-76.1) | 81.6 (71.9-89.1) | 45.1 (34.1-56.5) |
|  | 10 mo | 8.22 (6.22-10.86) | 11.83 (9.10-15.38) | 1.00 (0.78-1.30) | 96.1 (89.0-99.2) | 97.6 (91.6-99.7) | 56.3 (44.7-67.3) |
|  | 18 mo | 2.55 (2.02-3.20) | 2.55 (2.07-3.13) | 1.17 (0.92-1.49) | 81.3 (71.0-89.1) | 80.2 (70.3-88.0) | 55.3 (43.4-66.7) |
| **9V** | Birth | 3.02 (2.33-3.92) | 2.15 (1.61-2.87) | 2.64 (1.87-3.74) | 93.2 (81.3-99.0) | 82.9 (67.9-92.9) | 77.1 (59.7-89.6) |
|  | 2 mo | 1.21 (1.02-1.45) | 0.82 (0.69-0.96) | 0.66 (0.53-0.84) | 57.3 (46.4-67.7) | 47.3 (36.9-57.9) | 29.5 (20.6-39.7) |
|  | 3 mo | 1.64 (1.30-2.06) | 1.59 (1.23-2.06) | 0.36 (0.29-0.45) | 74.1 (63.5-83.0) | 61.8 (50.9-71.9) | 23.5 (15.0-34.0) |
|  | 4 mo | 1.29 (1.04-1.59) | 2.88 (2.33-3.56) | 0.26 (0.21-0.33) | 58.8 (47.6-69.4) | 85.6 (76.6-92.1) | 10.3 (4.8-18.7) |
|  | 9 mo | 0.89 (0.71-1.12) | 1.21 (0.99-1.47) | 0.36 (0.27-0.48) | 45.6 (34.3-57.2) | 63.2 (52.2-73.3) | 24.1 (15.4-34.7) |
|  | 10 mo | 5.94 (4.64-7.59) | 6.37 (5.13-7.92) | 0.87 (0.66-1.14) | 96.1 (89.0-99.2) | 97.6 (91.6-99.7) | 47.5 (36.2-59.0) |
|  | 18 mo | 1.50 (1.22-1.85) | 1.33 (1.11-1.59) | 0.82 (0.63-1.07) | 64.6 (53.0-75.0) | 61.6 (50.5-71.9) | 48.7 (37.0-60.4) |
| **14** | Birth | 4.73 (3.57-6.27) | 5.95 (4.48-7.92) | 5.21 (3.44-7.87) | 93.2 (81.3-98.6) | 97.6 (87.1-99.9) | 88.6 (73.3-96.8) |
|  | 2 mo | 1.91 (1.56-2.34) | 1.91 (0.58-2.32) | 1.58 (0.27-1.98) | 74.2 (63.8-82.9) | 74.2 (64.1-82.7) | 67.4 (57.0-76.6) |
|  | 3 mo | 1.78 (1.38-2.28) | 1.45 (1.18-1.80) | 0.80 (0.60-1.07) | 70.6 (59.7-80.0) | 60.7 (49.8-70.9) | 50.6 (39.5-61.6) |
|  | 4 mo | 2.54 (1.93-3.33) | 2.76 (2.14-3.57) | 0.63 (0.50-0.79) | 78.8 (68.6-86.9) | 80.0 (70.3-87.7) | 33.3 (23.6-44.3) |
|  | 9 mo | 2.84 (2.18-3.70) | 4.87 (3.77-6.27) | 0.75 (0.56-0.99) | 83.5 (73.5-90.9) | 90.8 (82.7-96.0) | 45.8 (34.8-57.1) |
|  | 10 mo | 7.88 (5.66-10.99) | 11.24 (8.57-17.74) | 1.24 (0.89-1.72) | 92.2 (83.8-97.1) | 94.0 (86.5-98.0) | 58.8 (47.2-69.7) |
|  | 18 mo | 2.31 (1.82-2.94) | 2.41 (1.90-3.06) | 1.89 (1.45-2.46) | 79.7 (69.2-88.0) | 76.7 (66.4-85.2) | 69.7 (58.1-79.8) |
| **18C** | Birth | 1.86 (1.41-2.47) | 1.55 (1.18-2.03) | 2.27 (1.56-3.32) | 75.0 (59.7-86.8) | 73.2 (57.1-85.8) | 77.1 (59.9-89.6) |
|  | 2 mo | 0.76 (0.62-0.93) | 0.49 (0.42-0.57) | 0.46 (0.37-0.57) | 38.2 (28.1-49.1) | 16.1 (9.3-25.2) | 25.3 (16.9-35.2) |
|  | 3 mo | 0.96 (0.74-1.23) | 0.98 (0.74-1.28) | 0.28 (0.22-0.35) | 49.4 (38.4-60.5) | 51.7 (40.8-62.4) | 10.6 (5.0-19.2) |
|  | 4 mo | 0.93 (0.74-1.16) | 2.33 (1.86-2.91) | 0.23 (0.18-0.29) | 49.4 (38.4-60.5) | 85.6 (76.6-92.1) | 10.3 (4.8-18.7) |
|  | 9 mo | 0.57 (0.45-0.72) | 0.72 (0.60-0.85) | 0.25 (0.20-0.33) | 31.6 (21.6-43.1) | 34.5 (24.6-45.4) | 8.4 (3.5-16.6) |
|  | 10 mo | 2.98 (2.36-3.76) | 3.70 (2.87-4.76) | 0.72 (0.55-0.93) | 81.8 (71.4-89.7) | 89.2 (80.4-94.9) | 38.0 (27.3-49.6) |
|  | 18 mo | 0.73 (0.58-0.93) | 0.71 (0.58-0.87) | 0.58 (0.46-0.74) | 36.7 (26.1-48.3) | 40.7 (30.2-51.8) | 34.2 (23.7-46.0) |
| **19F** | Birth | 6.25 (4.86-8.03) | 5.76 (4.51-7.36) | 7.61 (5.43-10.67) | 97.7 (88.0-99.9) | 95.1 (83.5-99.4) | 97.1 (85.1-99.9) |
|  | 2 mo | 2.84 (2.42-3.34) | 1.98 (1.69-2.32) | 1.89 (1.60-2.24) | 91.0 (83.1-96.0) | 79.6 (70.0-87.2) | 78.9 (69.4-86.6) |
|  | 3 mo | 2.98 (2.47-3.60) | 2.82 (2.29-3.47) | 1.03 (0.85-1.25) | 83.5 (73.9-90.7) | 86.5 (77.6-92.8) | 48.2 (37.3-59.3) |
|  | 4 mo | 2.31 (1.92-2.78) | 4.68 (3.90-5.63) | 0.94 (0.77-1.14) | 84.7 (75.3-91.6) | 96.7 (90.6-99.3) | 42.5 (32.0-53.6) |
|  | 9 mo | 3.06 (2.40-3.91) | 3.21 (2.56-4.03) | 2.25 (1.79-2.82) | 88.6 (79.5-94.7) | 81.6 (71.9-89.1) | 80.7 (70.6-88.6) |
|  | 10 mo | 7.78 (5.94-10.18) | 10.27 (8.35-12.62) | 3.11 (2.51-3.84) | 93.5 (85.5-97.9) | 100 (95.7-100) | 86.3 (76.7-92.9) |
|  | 18 mo | 5.50 (4.47-6.77) | 4.81 (4.06-5.70) | 4.18 (3.33-5.23) | 96.2 (89.3-99.2) | 97.7 (91.9-99.7) | 89.5 (80.3-95.3) |
| **23F** | Birth | 2.64 (2.00-3.48) | 2.04 (1.51-2.74) | 3.18 (2.16-4.68) | 88.6 (75.4-96.2) | 82.9 (67.9-92.9) | 77.1 (59.9-89.6) |
|  | 2 mo | 0.78 (0.65-0.94) | 0.59 (0.49-0.70) | 0.62 (0.51-0.75) | 37.1 (27.1-48.0) | 29.0 (20.1-39.4) | 31.6 (22.4-41.9) |
|  | 3 mo | 0.76 (0.59-0.98) | 0.56 (0.45-0.70) | 0.34 (0.27-0.44) | 43.5 (32.8-54.7) | 23.6 (15.2-33.8) | 14.1 (7.5-23.4) |
|  | 4 mo | 0.65 (0.51-0.83) | 1.87 (1.38-2.53) | 0.28 (0.23-0.36) | 37.6 (27.4-48.8) | 67.8 (57.1-77.3) | 13.8 (7.3-22.9) |
|  | 9 mo | 0.63 (0.47-0.84) | 0.79 (0.62-0.99) | 0.30 (0.23-0.39) | 38.0 (27.3-49.6) | 37.9 (27.7-49.0) | 18.1 (10.5-28.1) |
|  | 10 mo | 3.00 (2.13-4.24) | 5.55 (4.15-7.41) | 0.57 (0.46-0.71) | 76.6 (65.6-85.5) | 90.4 (81.9-95.8) | 32.9 (22.8-44.4) |
|  | 18 mo | 0.74 (0.57-0.95) | 0.84 (0.66-1.07) | 0.45 (0.35-0.58) | 43.0 (31.9-54.7) | 48.8 (37.9-59.9) | 23.7 (14.7-34.8) |
| **Non PCV7** |  |  |  |  |  |  |  |
| **2** | Birth | 1.96 (1.40-2.75) | 1.38 (0.97-1.97) | 2.17 (1.37-3.42) | 81.8 (67.3-91.8) | 63.4 (46.9-77.9) | 82.9 (66.4-93.4) |
|  | 2 mo | 0.56 (0.45-0.71) | 0.48 (0.37-0.60) | 0.49 (0.40-0.61) | 28.1 (19.1-38.6) | 28.0 (19.1-38.2) | 25.3 (16.9-35.2) |
|  | 3 mo | 0.21 (0.17-0.27) | 0.23 (0.18-0.28) | 0.22 (0.17-0.28) | 9.4 (4.2-17.7) | 6.7 (2.5-14.1) | 10.6 (5.0-19.2) |
|  | 4 mo | 0.28 (0.22-0.37) | 0.28 (0.23-0.33) | 0.22 (0.17-0.27) | 11.8 (5.8-20.6) | 7.8 (3.2-15.4) | 9.2 (4.1-17.3) |
|  | 9 mo | 0.44 (0.31-0.61) | 0.44 (0.35-0.54) | 0.37 (0.28-0.49) | 36.7 (26.1-48.3) | 20.7 (12.8-30.7) | 18.1 (10.5-28.1) |
|  | 10 mo | 3.54 (2.68-4.68) | 4.29 (3.28-5.61) | 4.30 (3.48-5.31) | 87.0 (77.4-93.6) | 90.4 (81.9-95.8) | 96.3 (89.4-99.2) |
|  | 18 mo | 2.05 (1.64-2.55) | 1.68 (1.28-2.20) | 2.51 (2.01-3.14) | 76.3 (65.4-85.1) | 69.8 (58.9-79.2) | 82.9 (72.5-90.6) |
| **5** | Birth | 2.90 (2.20-3.64) | 2.37 (1.81-3.09) | 3.87 (2.66-5.63) | 83.3 (68.6-93.0) | 87.5 (73.2-95.8) | 91.2 (76.3-98.1) |
|  | 2 mo | 1.30 (1.09-1.56) | 0.93 (0.79-1.10) | 1.04 (0.88-1.23) | 67.1 (56.0-76.9) | 48.3 (37.4-59.3) | 55.1 (44.1-65.6) |
|  | 3 mo | 0.88 (0.73-1.06) | 0.70 (0.58-0.85) | 0.48 (0.37-0.60) | 40.0 (29.2-51.6) | 41.7 (31.0-52.9) | 21.1 (12.5-31.9) |
|  | 4 mo | 1.25 (1.00-1.55) | 1.59 (1.28-1.96) | 0.56 (0.45-0.69) | 61.9 (50.7-72.3) | 72.8 (61.8-82.1) | 26.8 (17.6-37.8) |
|  | 9 mo | 1.16 (0.86-1.57) | 1.17 (0.90-1.53) | 0.99 (0.73-1.33) | 57.1 (43.2-70.3) | 51.8 (38.0-65.3) | 53.4 (39.9-66.7) |
|  | 10 mo | 3.78 (2.79-5.12) | 4.65 (3.42-6.32) | 2.18 (1.56-3.05) | 78.9 (66.1-88.6) | 89.5 (78.5-96.0) | 77.6 (63.4-88.2) |
|  | 18 mo | 1.64 (1.29-2.09) | 1.31 (1.02-1.67) | 1.36 (1.02-1.81) | 72.9 (59.7-83.6) | 56.7 (43.2-69.4) | 58.7 (43.2-73.0) |
| **7F** | Birth | 2.77 (2.03-3.78) | 2.18 (1.62-2.93) | 3.52 (2.42-5.13) | 79.5 (64.7-90.2) | 80.5 (65.1-91.2) | 91.4 (76.9-98.2) |
|  | 2 mo | 0.88 (0.72-1.08) | 0.80 (0.67-0.96) | 0.89 (0.74-1.07) | 47.2 (36.5-58.1) | 46.2 (35.8-56.9) | 47.4 (37.0-57.9) |
|  | 3 mo | 0.36 (0.30-0.43) | 0.40 (0.33-0.48) | 0.41 (0.34-0.49) | 8.2 (3.4-16.2) | 14.6 (8.0-23.7) | 17.6 (10.2-27.4) |
|  | 4 mo | 0.39 (0.32-0.48) | 0.39 (0.33-0.47) | 0.38 (0.31-0.46) | 11.8 (5.8-20.6) | 14.4 (7.9-23.4) | 12.6 (6.5-21.5) |
|  | 9 mo | 0.52 (0.38-0.73) | 0.55 (0.43-0.69) | 0.56 (0.44-0.72) | 30.4 (20.5-41.8) | 32.2 (22.6-43.1) | 33.7 (23.7-45.0) |
|  | 10 mo | 1.49 (1.14-1.95) | 1.84 (1.48-2.30) | 1.70 (1.35-2.13) | 64.9 (53.2-75.5) | 67.5 (56.3-77.4) | 77.5 (66.8-86.1) |
|  | 18 mo | 1.00 (0.77-1.30) | 1.03 (0.84-1.26) | 1.09 (0.87-1.38) | 48.1 (36.7-59.6) | 50.5 (39.0-61.0) | 57.9 (46.0-69.1) |
