# Supplementary material for: Safety and Immunogenicity of Neonatal Pneumococcal Conjugate Vaccination in Papua New Guinean Children: A Randomised Controlled Trial
Source: PLoS One. 2013 Feb 22;8(2):e56698. doi: 10.1371/journal.pone.0056698 (PMC3579820; doi:10.1371/journal.pone.0056698)
Supplement: File S1 — List of institutions and investigators comprising the Neonatal Pneumococcal Conjugate Vaccine Trial Study Team. (DOCX) [file pone.0056698.s006.docx]

**File S1 List of institutions and investigators comprising the Neonatal Pneumococcal Conjugate Vaccine Trial Study Team.**

*Papua New Guinea Institute of Medical Research*: E. Aemamero, M. Akunaii, H. Aole, E. Bilam, M. Dreyam, S. Eza’e, J. Francis, N. Fufu, E. Hasu, L. Helivi, G. Inapero, T. Jack, S. James, A Javati, H. Keno, W. Kirarock, I. Ko’ezo, M. Lai, A. Lapiso, A.M. Laumaea, S. Maraga, M. Martin, A. Michael, M. Michaels, A. Mope, P. Namuigi, B. Nivio, P. Ove, C. Opa, T. Orami, N. Paul, S. Phuanukoonnon, G. Poigeno, W.S. Pomat, J. Reeder (also Burnet Institute, Melbourne), G. Saleu, R. Sehuko, P. Siba, V. Siba, A. Sie, L. Sinke, J. Totave, B. Uro, G. Vengiau, L. Wawa’e, T. Wayaki, M. Yoannes

*Goroka Hospital*: Doctors J. Ande, J. Apa, D. Frank, W. Pame, N. Pomat. P. Keasu, A. Pikuri, H. Pok

*Telethon Institute for Child Health, Perth, Western Australia*: K.S. Alpers, C. Devitt, P.G. Holt, P. Jacoby, I. Laing (also University of Western Australia), D. Lehmann, M. Nadal-Sims, A. van den Biggelaar

*School of Paediatrics and Child Health, the University of Western Australia*: P.C. Richmond

*PathWest laboratory Medicine WA, Perth, Western Australia*: G. Chidlow, J. Harnett, D.W. Smith (also University of Western Australia)

*Curtin University of Technology*: M.P. Alpers

*Menzies School of Health Research*: A.J. Leach
