# Supplementary material for: Safety and Immunogenicity of Neonatal Pneumococcal Conjugate Vaccination in Papua New Guinean Children: A Randomised Controlled Trial
Source: PLoS One. 2013 Feb 22;8(2):e56698. doi: 10.1371/journal.pone.0056698 (PMC3579820; doi:10.1371/journal.pone.0056698)
Supplement: Protocol S1 — Trial Protocol. (DOC) [file pone.0056698.s007.doc]

**Application for Approval of a Research Project by the PNG MRAC**

**Project Title:**

Neonatal immunization with pneumococcal conjugate vaccine in Papua New Guinea

**Principle Investigators:**

Professor John Reeder

PNG Institute of Medical Research

PO Box 60, Goroka, EHP 441

Papua New Guinea

+675-732-1414

[john.reeder@pngimr.org.pg](mailto:john.reeder@pngimr.org.pg)

Associate Professor Deborah Lehmann

Division of Population Sciences

Telethon Institute for Child Health Research

PO Box 855, WA 6872

+61-8-9489 7755

[deborahl@ichr.uwa.edu.au](mailto:deborahl@ichr.uwa.edu.au)

**Collaborating Investigators:**

Professor Patrick Holt

Telethon Institute for Child Health Research

Division of Cell Biology

PO Box 855 , WA 6872

+61-8-9489 7838

[patrick@ichr.uwa.edu.au](mailto:patrick@ichr.uwa.edu.au)

Dr Peter Richmond

Dept. Paediatrics,

University of Western Australia

Princess Margaret Hospital for Children,

Roberts Rd., Subiaco, WA 6008

Mr William Pomat

PNG Institute of Medical Research

PO Box 60, Goroka, EHP 441

Papua New Guinea

(Currently studying in Perth with Dr Richmond)

**Plain language Summary**

Worldwide, one million children die annually of pneumococcal (Pnc) disease and pneumonia is the primary cause of mortality in Papua New Guinean children. Many die in early infancy and babies may benefit from immunization with a Pnc conjugate vaccine (Prevenar™) at birth. The National Health Plan 2001-2010 calls for investigation of the feasibility of Pnc vaccines for PNG.

The Papua New Guinea (PNG) Institute of Medical Research, Telethon Institute for Child Health Research and the Department of Paediatrics, University of Western Australia will collaborate to examine very closely the safety of this approach, particularly with regard to impact on the development of immunity and response to other vaccines given to infants. This study will also provide a unique opportunity for training of PNG and Australian scientists in both countries, transfer of state-of-the-art immunological technology and stimulate further collaborations on respiratory infections in the region.

**Technical Summary**

Infants in Papua New Guinea (PNG) are at extremely high risk for invasive pneumococcal (Pnc) disease. Similar to other high-risk populations, PNG infants have neonatal onset of dense respiratory tract Pnc colonisation, which may have long-term effects on the development of protective immunity. Pnc conjugate vaccines (PCV) that are efficacious in American and African infants need evaluation in this high-risk PNG population under the national accelerated 1-2-3 month schedule. In order to obtain the earliest possible protection against invasive disease, achieve optimal coverage and reduce burden of early carriage, neonatal PCV immunization needs to be considered. This study in the PNG highlands will enrol 312 infants at birth, who will be randomised to receive PCV either at 1-2-3 months or 0-1-2 months of age or receive only routine immunizations (controls). Blood samples will be taken at birth-2-3-4 months of age, pre- and post-Pnc polysaccharide booster at 9-10 months of age (to assess immune memory) and at 18 months at study completion. Carriage will be assessed weekly for the first month of life and at regular intervals thereafter. There will be ongoing surveillance for respiratory and other diseases throughout the study. In addition to serotype-specific IgG, we will examine IgG avidity, IgG subclasses, mucosal IgA and T-cell cytokine responses to PCV and Pnc protein antigens. To ensure immunological safety, particularly for neonatal PCV, immune responses to concomitant vaccines and viral and environmental antigens will also be examined as well as overall T-cell maturation.

This study will provide proof of principle of the safety and immunological feasibility of neonatal PCV immunization, which is essential before progression to larger-scale studies in high-risk populations. This study will also examine the effect of early carriage on the development of systemic and mucosal immunity to Pnc infections and the impact of early PCV on carriage. It will further our understanding of the basic immunological mechanisms underlying conjugate vaccine responses during the critical neonatal period, and provide insight into the interactions between the developing T‑cell system and vaccines, which occur in these infants against a background of intense microbial stimulation. These studies are crucial for the optimal use of current vaccines and the development of new vaccines, for Pnc and other pathogens. This project will bring together the necessary expertise, innovative methodology and laboratory facilities in PNG and Australia to conduct the study in order to reduce this major burden on child health.

**Aims of the Study**

In Papua New Guinea (PNG), there is an early onset of dense bacterial carriage in the upper respiratory tract (URT) and a high pneumococcal (Pnc) disease burden in the first year, especially under 6 months of age. This study aims to investigate a number of important, unanswered questions that are critical to evidence-based vaccine intervention:

1. Is immunization with pneumococcal conjugate vaccine (PCV) safe and immunogenic in PNG infants, when administered according to the standard immunization schedule in PNG (1-2-3 months)?
2. Does neonatal immunization with PCV provide a more favourable antibody response in early childhood than immunization starting at 1 month of age, without compromising safety?
3. Does neonatal or early infant immunization with PCV induce immunologic memory to the relevant Pnc polysaccharides?
4. Does neonatal or early infant immunization with PCV affect nasopharyngeal carriage of vaccine and non-vaccine serotypes?
5. What is the impact of early colonisation with vaccine serotypes on antibody responses (systemic and mucosal) to neonatal or early infant immunization with PCV?
6. Is the response of PNG infants to Pnc antigens governed by developmental factors controlling postnatal maturation of immune competence?
7. Is there evidence that early carriage of Pnc leads to tolerance or impaired responses to vaccine or non-vaccine Pnc antigens?
8. With respect to immunological safety of neonatal immunization with PCV in PNG infants :
9. Does neonatal immunization with PCV interfere with humoral and cellular immune responses to concomitant vaccines (diphtheria toxoid, tetanus toxoid and measles)?
10. Does PCV interfere with normal maturation of the immune system, in particular Th1 functions which mature rapidly during infancy?
11. Does PCV reduce the incidence of respiratory morbidity in PNG infants in the first year of life?

In addition, the study will provide a more detailed immunologic evaluation of pneumococcal polysaccharide vaccine (PPV) in the first year of life and will re-establish demographic surveillance within a field site ready for an efficacy study of neonatal immunization, if justified by the results of the present study, or for the evaluation of other candidate pneumococcal vaccines.

**Background: importance of the health problem and work which has led to the project**

# **i) Pneumococcal disease: a major global health problem of young children**

It is estimated that 2.1 million children die annually from pneumonia – ‘more than HIV, tuberculosis and malaria combined’ (1) – the majority in the developing world; *Streptococcus pneumoniae* (pneumococcus; Pnc) is responsible for approximately one million of these deaths (2). Pnc is also a leading cause of meningitis, bacteraemia and otitis media (OM). Invasive pneumococcal disease (IPD) is a major cause of morbidity in other indigenous populations as well (3, 4): in Central Australian children <5 years of age, the annual incidence rate of IPD is 935/10^5^ in Aboriginal children compared to 87/10^5^ in non-Aboriginal children (4). Young infants are at particularly high risk of Pnc disease. In young infants in 4 developing countries, Pnc was the most common cause of serious infection under 1-2 months and of meningitis under age 3 months (5). Interventions to prevent this mortality and morbidity in infants are urgently needed. The Global Alliance for Vaccines and Immunization (GAVI) has identified protection of infants from early IPD by neonatal PCV immunisation as a priority research area.

# Burden of pneumococcal disease in Papua New Guinean children

Young children in PNG are at particularly high risk for serious Pnc infections. Pneumonia is the most common reason for childhood hospitalisation and mortality in PNG. In the Asaro Valley, Eastern Highlands Province (EHP), where we propose this study, mortality rate for acute lower respiratory infections (ALRI) in infants is 2500/10^5^/annum; 56% of all ALRI deaths in children <5 years occur under age 6 months (6). Pnc accounts for 46% of bacteraemic pneumonia (7), with 26% and 63% of children aged <3 and <6 months, respectively. Pnc was the most common pathogen isolated in a recent audit of paediatric deaths (8). Pnc also causes ~45% of bacterial meningitis with 69% of cases <6 months (9). Pnc penicillin resistance is a concern with the recent emergence of multi-drug resistant strains (10).

Pnc is also a leading cause of mucosal infections such as OM, resulting in chronic suppurative OM and hearing loss (3, 11). In one study in the Asaro Valley, CSOM was seen in 17% of village children aged 6-11 months (Lehmann, unpublished observations). The high adult rate of IPD in high-risk populations including PNG (4, 12) reflects ongoing risk factors such as overcrowding but may also be due to an impaired development in long-term immunity to Pnc infections.

**ii) URT carriage of Pnc in high-risk populations and association with disease**

Early onset of dense URT bacterial colonisation in children living in developing countries and other indigenous children is associated with increased risk of disease (11, 13). This early carriage may also lead to immunological hyporesponsiveness to Pnc antigens leading to persistent susceptibility to disease (11). In the Asaro Valley, all children acquire Pnc by 3 months, 60% during the neonatal period, with a median age of acquisition of 17 days (14) and simultaneous carriage of multiple Pnc serotypes is common (15). Pnc carriage remains high throughout childhood. Serotypes contained in PCV (4, 6B, 9V, 14, 18C, 19F & 23F) account for 60% of carriage isolates (16). Thus non-PCV serotypes are more commonly carried (40%) than in low-risk populations, eg 23% in USA (17). In a Western Australian study, 41% of Aboriginal children and 12% of non-Aboriginal children are colonised with Pnc by the age of 2 months (18) compared to <10% in USA infants (19). Furthermore, in PNG we have found that IPD is associated with high acquisition rates and high frequency and prolonged duration of carriage (13) indicating that both systemic and mucosal immunity may be impaired.

**iii) Natural acquisition of immunity to pneumococcal infections**

Despite the large global burden of Pnc disease, relatively little is known about the development of natural Pnc immunity in humans. Protection against IPD is via antibody-mediated opsonophagocytosis. Pnc CPS and outer membrane proteins (OMPs) are the most important targets of protective immune responses. CPS is an important virulence factor as it enables the Pnc to survive in blood. 20 of the 90 immunologically distinct Pnc serotypes commonly cause disease. Antibodies directed at the capsule are serotype-specific with some cross reactivity within serogroups (e.g. 6A & 6B). Serotype distribution varies according to site of isolation (e.g. nose, blood or CSF), type of disease (OM or IPD), geographic location and age. This is important when considering potential benefits of Pnc conjugate vaccines (PCV) as the serogroups included in the licensed 7-valent PCV (Prevenar™) account for 89% of IPD in children in USA but only 43% in Asia (20). In PNG, PCV covers 52% of childhood bacteraemic pneumonia (7).

Most work on the development of Pnc-specific immunity has focused on serum anti-capsular IgG that correlates with serotype-specific opsonophagocytic activity (OPA). Vaccine efficacy studies have confirmed their role in protection against serotype-specific invasive disease. Passively acquired maternal IgG antibodies offer some early protection for infants although duration of maternal antibodies is shorter in developing countries such as PNG (21). Naturally acquired CPS antibodies are predominantly IgG_2_ subclass and gradually increase following exposure through carriage but do not reach adult levels against most serotypes until late childhood. The ontogeny of serum antibody responses to different Pnc CPS varies considerably, with development of CPS IgG following carriage with some immunogenic serotypes (3, 11A, 14) as early as 6 months of age, but not before 2 years of age for less immunogenic serotypes (6B, 19F, 23F) (22, 23). In PNG infants, despite frequent and heavy carriage, serum anti-capsular IgG levels remain low for the first 2 years of life (24) and children who develop pneumonia have even lower antibody levels (25). Pnc carriage is also associated with mucosal IgA responses to homologous CPS from as early as age 6 months, starting earlier than serum CPS responses (26). Mucosal antibodies are likely to be important in protection against mucosal and systemic infections and carriage but there are few data in high-risk infants.

### iv) Vaccines for the prevention of pneumococcal disease

The recognition that immunity to IPD correlated with anti-CPS antibodies led to the development of a Pnc polysaccharide vaccine (PPV) containing CPS of 23 serotypes. This vaccine is immunogenic and protects adults against IPD. However, as CPS is a T-cell independent antigen, its immunogenicity is reduced in infants, with low avidity antibody that may not be functional. It does not prime for immunologic memory, so protection is short-lived. PPV does not protect against carriage and offers limited protection against OM (27). The least immunogenic serotypes in PPV are responsible for most disease in young children in the first world. However, in the developing world, the more immunogenic serotypes account for a higher proportion of IPD and many elicit protective responses at an early age (24, 28-30).

# Studies of pneumococcal polysaccharide vaccine (PPV) in PNG children

In view of the high rates of IPD in young PNG children, we investigated early use of PPV and demonstrated that PPV given at 6 months to 5 years of age prevents death from ALRI (efficacy 59% and 50% in children aged <5 and <2 years, respectively) with 19% reduction in total mortality in the vaccinated group (31). In young children, PPV affords some protection against severe but not mild ALRI (32). Serotype-dependent maturation of antibody responses support efficacy data (24, 31) with 2-fold rises in IgG titres one month post-PPV at ages 5, 6, 9 and 12 months for serotypes 2 ,7F, 23F and 5, respectively, but only after 18 months for PCV serotypes 6B, 14 and 19F. An effectiveness study of PPV further confirms our efficacy data with a hazard ratio of 0.42 for death at age 12-23 months in immunized compared to unimmunized children (33). These findings are at variance with results from PPV trials in industrialised countries (34) but the discrepancies may be explained by 1) the high incidence of severe disease and mortality in PNG, thus having the power to demonstrate efficacy, 2) common serotypes causing disease in PNG are immunogenic in PPV and 3) PPV prevents invasive disease but not mucosal infections. Our findings to date support the use of PPV at age 9 months in our proposed study; furthermore, more detailed immunologic assessment of PPV responses in PNG children is indicated, in particular the quality of antibody produced, as antibody avidity influences the functional activity of anti-CPS antibodies (35).

# Pneumococcal conjugate vaccines

The limitations of polysaccharide vaccines can be overcome by conjugating CPS to a carrier protein such as modified diphtheria toxin (CRM_197_) used in *Haemophilus influenzae* type b (Hib) conjugate vaccines (36). Carrier protein-specific T-cells provide help for polysaccharide-specific B-cells via cytokines and co-stimulatory molecules. This results in a T-cell-dependent CPS antibody response characterised by increased immunogenicity in infants, affinity maturation resulting in high avidity functional antibody and induction of memory with long-term protection. The success of Hib conjugate vaccines has led to the development of an immunogenic and efficacious PCV (37). Despite the success of conjugate vaccines the optimal carrier-specific T-cell cytokine response for B-cell stimulation and priming for memory is not known. Indeed, carrier-specific suppression may occur with some combination vaccines where increasing doses of carrier protein results in impairment of antibody response to all vaccines containing that protein (38). This may limit the number of serotypes that can be included in PCV. The avidity of CPS IgG also varies with the carrier proteins used (39). Preliminary studies of T-cell responses to carrier proteins in adults given PCV suggest that predominantly Th2 cytokines are involved in the carrier protein response (40).

There is only one licensed PCV, Prevenar™ (containing 7 serotypes conjugated to the CRM_197_ carrier protein), which prevented 97% of IPD due to vaccine serotypes when given at 2, 4, 6 and 12-15 months of age in US infants (37). There was also a 21% reduction of chest x-ray (CXR)-proven pneumonia (41) and reductions in OM and operations for grommets. A similar PCV containing 9 serotypes (9V-PCV; addition of types 1 and 5 to Prevenar™) is highly immunogenic at 6-10-14 weeks of age in South African (SA) infants and reduces carriage of vaccine serotypes (42). There was no reduction in antibody responses to concomitant diphtheria, tetanus or Hib conjugate vaccines (43). Significant antibody responses (>0.5μg/ml) were seen after one dose at 6 weeks of age in 34-98% of infants (74-99% after 2 doses), depending on serotype, with significantly higher antibody levels for 7 serotypes compared to placebo controls (43). Responses to 3 doses of PCV seen in SA infants were higher for 6 of 7 serotypes compared to US infants receiving the 7V-PCV vaccine (37), suggesting natural boosting by Pnc carriage in the African children after priming by 9V-PCV. Preliminary results from the SA trial of 9V-PCV given to 30000 infants indicate efficacy of 85% (95% CI 32-98%) against vaccine-serotype IPD and 25% (4-41%) against CXR-proven pneumonia (all causes), in a high-risk population under a 6-10-14 week schedule with no booster dose (Klugman, pers comm). There are no studies evaluating PCV with the accelerated PNG schedule at 1-2-3 months of life. In addition carriage rates in PNG infants are substantially higher and earlier than in SA infants, which may have beneficial or negative effects. Careful evaluation of the 1-2-3 month schedule is required and it may also be applicable for other high-risk populations such as indigenous Australian infants.

PCV vaccines induce mucosal immunity with serotype-specific salivary IgA responses in infants (44). Responses are modest after the primary series; however, evidence of mucosal priming for memory is seen after booster doses and may be important in protection against carriage and OM. A number of studies have confirmed that PCV reduces nasopharyngeal carriage of vaccine serotypes (38, 42) and may result in reduced disease in unvaccinated individuals or herd immunity as was seen after Hib conjugate vaccines. PCV also prevented 57% of OM episodes due to vaccine serotypes in Finnish infants (45) but the 34% increase in OM due to non-vaccine serotypes highlights the need for careful follow-up in intervention studies. To date there has been no increase in IPD due to non-vaccine serotypes following PCV, but this is a potential risk in a high incidence population such as PNG infants where non-vaccine serotypes (e.g. 2 and 7F) more commonly cause IPD. Before introducing PCV routinely in such populations, it is necessary to monitor the serotypes of Pnc carriage and IPD and the frequency of ALRI following PCV.

PCV is now licensed in North America, Europe and Australia but its cost ($A100/dose) has limited its routine use to USA, Canada and France and Australian indigenous infants. PCV primes for memory responses to PPV (44, 46) and PPV is used as booster in Australian indigenous children. This not only boosts PCV serotype responses but also protects against disease due to non-PCV serotypes. This is important because of possible serotype replacement and the burden of IPD due to non-PCV serotypes in high-risk populations. A study of alternative regimens of Pnc vaccines (1-2 doses of PCV followed with a booster of PPV), which might be affordable, and logistically more appropriate, will start soon in Fiji (Mulholland, pers comm). Inclusion of a neonatal dose is awaiting results of immunological safety studies. Our study will provide this information and additional data on responses to 1 and 2 doses of PCV.

# **v) Neonatal immunization**

As newborns are particularly susceptible to infectious diseases, the World Health Organization (WHO) has identified neonatal vaccination as a research priority, in particular the quality of Th1 versus Th2 responses to existing vaccines (see vii below) and induction of appropriate systemic and mucosal immune responses. In neonatal immunization, assessment of antibody alone may be misleading as maternally derived antibody interferes with vaccine-induced antibody production but does not inhibit T-cell responses and priming of memory B-cells (36). Neonatal hepatitis B and BCG immunization is safe and effective in developing countries with high coverage. Data on neonatal immunization with polysaccharide-protein conjugate vaccines is limited to ~200 neonates given Hib conjugate vaccines, which were well tolerated with evidence of priming after a single dose (47, 48). Neonatal immunization with Hib conjugate vaccines with tetanus and CRM_197_ carrier proteins does not affect Hib or carrier protein antibody responses post-primary immunization, but the Hib-meningococcal OMP conjugate vaccine (PRP-OMP) is associated with a decreased response to subsequent PRP-OMP vaccination, suggesting induction of hyporesponsiveness (49). The mechanism for this is unclear as there was no evaluation of T-cell responses. In view of the high mortality from IPD in infancy and to achieve optimal vaccine coverage, an expert WHO committee has recommended evaluation of PCV neonatal immunization (50). Prior to larger-scale efficacy studies of neonatal PCV in high-risk groups, the safety and immunological feasibility of this approach must be established. In addition to serum responses to Pnc antigens, assessment of mucosal Pnc antibody is important, given the role of IgA in prevention of Pnc carriage and mucosal infections, including pneumonia. Neonatal PCV vaccination must also consider potential effects on postnatal maturation of the immune system, in light of recent concerns regarding non-specific effects of immunization on mortality in African children (51) – increased mortality with DTP in African but not PNG children (33) but reduced mortality with measles and BCG vaccines in both populations. These findings highlight the need for detailed evaluation of non-specific effects of new vaccines and new schedules on the maturation of humoral and cellular immunity during infancy (see vii/viii below).

**vi) Immune responses to pneumococcal outer membrane proteins**

Individuals who are colonised with pathogenic strains may not mount antibody responses against CPS but do not develop disease indicating that other factors, such as antibodies and cellular immune responses against Pnc OMPs, may play a role in protection (52). Candidate protein vaccines evaluated in animal models showed protection against carriage (pneumococcal surface adhesin A: PsaA), against sepsis (pneumolysin: Ply) and against both carriage and invasive disease (pneumococcal surface protein A: PspA) (52). Studies in humans support these findings. Serum anti-PsaA IgG is lower in Gambian infants who are Pnc carriers (53). In adult Pnc pneumonia, anti-Ply IgG protects against bacteraemia (54). Anti-PspA IgG protects adults against Pnc colonisation after direct challenge (55) and low levels of PspA IgG are found in children with IPD (56).

The development of immunity to Pnc OMPs has been studied in Finnish infants in relation to carriage and OM (57, 58). OMP antibodies are induced by carriage and OM in young children in serum (57) and saliva (58). The ontogeny of antibody responses varied between proteins: PsaA antibodies rise rapidly to adult levels by 6 months of age, Ply antibodies increase by 12 months but are still below adult levels at 2 years and PspA antibodies are still low at 2 years of age in both serum and saliva (57, 58). Early appearance of salivary OMP antibodies by 6 months of age was associated with an increased risk of subsequent OM suggesting that early exposure may have resulted in a maladaptive response that is not protective (26). There are no published data on the ontogeny of T-cell immunity to Pnc OMPs in children. Variations in the development of T-cell immunity to Pnc proteins may influence systemic and mucosal antibody responses and priming for memory, which is critical in protection against Pnc infections. Our study will provide better understanding of T-cell responses to Pnc OMPs, their influence on humoral responses and their relationship to carriage and overall maturation of the immune system. This will be critical for the development of protein-based vaccines.

**vii) Key developmental issues relevant to responsiveness to microbial antigens during infancy**

It is evident from animal and human studies that a number of control mechanisms regulating host defence are functionally immature at birth, thus compromising the efficiency of innate and adaptive immunity during early infancy. One of the manifestations of this developmental deficiency is the relative polarisation of T‑helper (Th) cell activity towards preferential expression of Th2 cytokines, a process that is believed to play a key role in protection of the placenta against the toxic effects of Th1 cytokines such as TNFα and IFNγ (59). This Th2 polarisation is maintained via a series of interlocking cellular and molecular mechanisms, which include attenuation of the capacity of immature antigen presenting cells to elaborate Th1‑stimulatory signals (60) such as IL‑12 (61, 62), and hypermethylation of CpG sites in the proximal promoter of the IFNγ gene in neonatal CD4^+^ Th‑cells (63) which attenuates IFNγ gene transcription. These and related control mechanisms cumulatively maintain the Th2 polarity of adaptive immune function into postnatal life, and we have demonstrated in forerunner studies in human infants that this manifests as the initial selective expression of Th2‑polarised immunity to vaccines such as DTPa (64).

We have additionally demonstrated that the kinetics of postnatal development of the capacity to produce the key Th1 cytokine IFNγ is highly variable between individuals, and moreover that expression of stable Th1‑polarised memory to DTPa vaccine is directly linked to this developmental process (65). There is increasing evidence that under appropriate conditions of priming, neonatal T‑cells have the intrinsic capacity to generate significant Th1 responses, but have higher stimulation thresholds than their adult counterparts (66). In support of this proposition, it has been demonstrated that in contrast to the DTPa vaccine, immunization with potent Th1‑trophic BCG vaccine elicits strong primary Th1 responses in African infants (67).

It is also evident that variations in the kinetics of postnatal development of Th1 “competence” may also underlie variations in susceptibility to infections in infancy. In indirect support of this proposition, we have recently demonstrated that expression of an immunological “footprint” of RSV infection (i.e. RSV‑specific cytokine responses by PBMC) was most frequent in infants who were slowest in developing postnatal IFNγ response capacity (65). Severity of symptoms following infant RSV infection has also been linked with an attenuated IFNγ response (68-70), and similar suggestions have been raised in regard to IL‑12 (71).

In summary, during infancy the capacity to generate stable Th1 immunity to vaccination, and to mount effective cellular immune defence against pathogenic microorganisms, is (a) highly variable between subjects, and (b) is dependent upon covert developmental processes underlying transition of the immune system from the foetal-like Th2‑polarised state, to the more balanced state typical of adults. This developmental process is driven by microbial stimulation (both pathogens and commensals) from the extrauterine environment, and involves a range of specialised receptors (notably CD‑14 and the TOLL family). The genes encoding these receptors are known to be highly polymorphic, and moreover the frequency of functional polymorphisms in these genes varies markedly between populations from temperate versus tropical environments (Lesouef, personal communication). Currently, virtually all the information relevant to these important vaccine-related issues is derived from studies on Caucasian infants, and the degree to which such studies are predictive of vaccine responses in paediatric populations such as those in PNG is not known. Indeed one would expect, with the heavy load of diverse pathogens to which the young infant is exposed in these populations, that the vaccine responses and the maturation of the immune system would be very different.

**viii) Early bacterial colonisation and susceptibility to invasive infection: significance of neonatal “tolerance” phenomena**

It has recently been recognised that the developmental mechanisms underlying postnatal acquisition of balanced Th1/Th2 immunity are also central to the phenomenon of neonatal tolerance. The latter process involves the predilection of the immature immune system to respond to initial antigenic challenge via the development of specific unresponsiveness. However, the readout systems employed to define neonatal tolerance almost exclusively measure Th1 immunity, and it has recently been shown that ostensibly “tolerised” neonates in fact manifest covert Th2‑polarised immunity, which is preferentially primed at this age due to the pre‑existing Th2 bias in the immature immune system. Importantly, as well as favouring the manifestation of Th2‑polarised immunity during primary immune responses in infancy, this can also result in development of Th2‑biased Th‑memory which is revealed by re‑challenge in adult life (72).

To date, these experimental neonatal tolerance systems have focused upon responses to inert antigens. However, a recent study on murine RSV infection indicates that identical principles apply to immunity to infection during the immediate postnatal period (73, 74). In particular, if initial RSV infection occurs very early during infancy it triggers Th2‑polarised primary immunity, and also generates Th2‑polarised memory which is preferentially recalled following reinfection in adulthood. Moreover, the older the mice are at the time of initial RSV exposure, the greater is the Th1 component in both their primary and (subsequently) secondary immunity, and eradication of the infection is correspondingly more efficient.

In the context of this project, we hypothesise that similar principles are applicable to resistance mechanisms relevant to bacterial infection, including pneumococci. In particular, studies from several centres (11, 16) suggest that colonisation of respiratory mucosal surfaces with Pnc during early infancy may compromise capacity to subsequently develop efficient long-term immunity to IPD. This raises the possibility that “neonatal tolerance” to polysaccharide and/or protein antigens associated with Pnc may develop in response to early colonisation during infancy. By analogy with the findings above relating to RSV, the Th2 component of host immunity may also dominate responses to Pnc during early infancy. It is feasible that this response phenotype may contribute to the development of a stable (non‑inflammatory) relationship between Pnc and the upper respiratory mucosa, in disease-free subjects who carry this organism as a commensal. This has a precedent in findings demonstrating the key role of Th2‑associated IL‑10 in maintaining stable host-commensal relationships in the gastrointestinal mucosa (75), and we propose to ascertain whether similar form(s) of T‑cell immunity are associated with persistent Pnc carriage in PNG infants.

Additionally, based upon experience from animal model systems, we believe it is likely that Th2 immunity of this nature has the potential to compromise the development and/or expression of Th1 immunity in response to PCV or Pnc carriage and indeed during invasive Pnc disease. Consequently, an important element in the study described below will be comparison between the responses to PCV in children colonised with Pnc at different stages during infancy, focusing in particular on subjects colonised during the first two weeks of life, when the Th2 polarity of their immune systems should be maximal.

Rationale for the proposed study

Infants in PNG are at extremely high risk for IPD with a substantial burden of disease in the first 6 months of life. Similar to other high-risk populations, PNG infants have early onset (<1month of age) of dense URT colonisation with multiple strains of Pnc, which may have long-term effects on the development of immunity to Pnc disease. Pnc conjugate vaccines that are efficacious in American and African infants need evaluation in this high-risk population. In order to obtain the earliest possible protection against invasive disease, achieve optimal coverage and reduce burden of early Pnc carriage, a schedule including neonatal immunization needs to be considered. This study will provide proof of principle of the safety and immunological feasibility of neonatal PCV immunization which is essential before progression to larger-scale studies in high-risk populations, and will also examine the effect of early carriage on the development of systemic and mucosal immunity to Pnc infections and the impact of early PCV on carriage. It will further our understanding of the basic and humoral immunological mechanisms underlying conjugate vaccine responses during the critical neonatal period, and in particular will provide new insight into the interactions between the developing T‑cell system and the vaccine. These studies address crucial issues related to the optimal use of current infant vaccines and the design and development of new vaccines, for Pnc and other pathogens. The PNG Institute of Medical Research (PNGIMR) has 30 years experience in carrying out community-based multidisciplinary research, particularly in respiratory diseases, and is a designated WHO Collaborating Centre for Research and Training on Acute Respiratory Infections. The Institute for Child Health Research (ICHR) has unique expertise in large-scale cohort studies related to perinatal immune function. Bringing these two groups together in the project will create an exciting opportunity for a comprehensive and coordinated attack on this major problem of child health.

**Experimental design and methods to be used in investigating these scientific questions**

###### i) Overall study design

# We propose to conduct an open, randomised controlled trial (RCT) of reactogenicity and immunogenicity of a 7-valent Pnc-CRM_197_ conjugate vaccine (Prevenar™) in 312 infants in the Asaro Valley, PNG, where extensive population- and hospital-based research has been conducted into the epidemiology and prevention of ALRI (6). Following ethical approval, principally by the PNG Medical Research Advisory Committee (MRAC) and also by the respective institutional review boards in Australia, demographic surveillance will be established in villages of Lowa Census Division (CD). Pregnant women will be enrolled in these villages and also at the Goroka Hospital antenatal clinic. Following delivery, infants will be randomised to one of three equal-sized groups to receive: 1) PCV at ages 0-1-2 months, 2) PCV at ages 1-2-3 months and 3) no PCV in addition to routine infant immunizations and PPV at age 9 months. Table 1 shows the vaccines given, samples collected and surveillance maintained. Nasopharyngeal Pnc colonisation will be assessed frequently for the first 4 weeks of life and at regular intervals thereafter to look for effects of early colonisation on subsequent immune responses to PCV and vice-versa. Infants will undergo comprehensive immunological evaluation for systemic and mucosal antibody responses, T-cell-specific cytokine responses, induction of immunologic memory for PCV CPS and antibody avidity maturation. We will also examine kinetics of postnatal maturation of humoral and T-cell immunity over time.

### Table 1. Neonatal Immunization with Pnc Conjugate Vaccine Study Outline

|  | Birth | 1 -3wk | 1mth | 2mths | 3mths | 4mths | 6 mths | 9mths | 10mths | 18mths |
| --- | --- | --- | --- | --- | --- | --- | --- | --- | --- | --- |
| Group 1 Neonatal PCV | HepB, BCG, OPV, PCV |  | DTP/Hib, HepB, OPV, PCV | DTP/ Hib, OPV, PCV | DTP/ Hib, OPV, HepB |  | Measles | PPV Measles |  |  |
| Group 2 Infant PCV | HepB, BCG  OPV |  | DTP/Hib, HepB, OPV, PCV | DTP/Hib, OPV, PCV | DTP/Hib, OPV, HepB, PCV, |  | Measles | PPV Measles |  |  |
| Group 3 Control | HepB, BCG  OPV |  | DTP/Hib, OPV, HepB | DTP/Hib, OPV | DTP/Hib, OPV, HepB |  | Measles | PPV Measles |  |  |
| Nose Swab |  | weekly | X |  | X |  |  | X |  | X |
| Saliva 1-2 ml |  |  | X | X | X | X |  | X | X | X |
| Serum 2-4 ml | **X*** |  |  | X | **X** | **X** |  | **X** | **X** | **X** |
| PBMC 3-5ml | **X*** |  |  |  | **X** |  |  | **X** | **X** | **X** |
| Morbidity surveillance |  |  |  |  |  |  |  |  |  |  |

HepB: hepatitis B vaccine; PCV: 7-valent Pnc-CRM_197_ conjugate vaccine; OPV: oral polio vaccine; DTP: diphtheria-tetanus-whole-cell pertussis; Hib: *Haemophilus influenzae* type b tetanus toxoid conjugate vaccine (PRP-T); PPV: 23-valent Pnc polysaccharide vaccine

***** Cord blood sample: total 10ml taken for PBMC (peripheral blood mononuclear cells)

### ii) Field methods

# Study setting

The Asaro Valley lies 6° south of the equator and people live between 1500 and 1900 metres above sea level. Goroka town (1990 census: population 18000) is the provincial capital. PNGIMR headquarters, laboratories and small clinic are located next to Goroka Hospital, the only tertiary hospital in the area, where most women from the study area deliver and where there are daily antenatal clinics. Living standards vary widely in the town. The Lowa CD (1990 census: population 29000) is adjacent to Goroka town; people live in villages (accessible by 4WD vehicles on unsealed roads) and are primarily subsistence farmers.

A network of village reporters carried out demographic surveillance by house-to-house-visiting on a random sample of the population of the Asaro Valley, including Lowa CD and Goroka town, for epidemiological studies of respiratory and enteric diseases (6). In the late 1980s, infant mortality and crude birth rates in Goroka were 32/1000 livebirths and 43/1000/annum, respectively; equivalent figures for Lowa CD were 45 and 31. More than 70% of women delivered in hospital (DL unpublished data); now, supervised delivery rates are likely to be higher. Over 1000 women attend the antenatal clinic at Goroka Hospital annually. D Lehmann and the field supervisor G Saleu are well-known to local residents and have intimate knowledge of the area as well as the methodology from these earlier studies. Population-based HIV prevalence data are not available; however, no positive cases were found during a recent survey of 220 sex workers in Goroka.

# Demographic surveillance and recruitment of study participants

After informing communities about the study, a house-to-house census will be undertaken in those villages previously under surveillance in Lowa CD. Surveillance by local reporters will identify pregnant women, ensure close monitoring for safety of neonatal immunization for 18 months post-vaccination and re-establish a population base for future studies including an efficacy trial of neonatal PCV if appropriate. If necessary, surveillance will be extended to neighbouring villages to include a total population of more than 3000 in order to identify 100 pregnant women annually. A nurse will also recruit women through antenatal clinics at Goroka Hospital to enrol an equal number of urban children. In view of the intensive monitoring schedule, no more than 2 urban and 2 rural mothers will be recruited weekly. Data on URT carriage rates in the urban population are not available; however, we might expect later onset of URT Pnc carriage in babies living in the less crowded, more hygienic urban living conditions. By enrolling equal numbers of participants from urban and rural areas we can investigate the effect of early onset of carriage on subsequent immune responses. Following enrolment at antenatal clinic, a home visit will be done to collect social and demographic data on the entire household (for comparison between urban and rural areas). This will help re-establish an urban population base for future trials. Weekly surveillance of all households in enlisted villages and of enlisted families in Goroka will continue for the entire study period for safety monitoring, identification of potential new participants, registration of deaths and migrations in and out of the area. Each person under demographic surveillance is assigned a unique identifying number used to link all data.

# Enrolment of study participants and consent

Once staff have been trained and a village is under weekly surveillance, pregnant women can be enrolled, while continuing to expand the demographic base. Informed consent will be sought antenatally. The standard process for obtaining informed consent for studies in PNG, as used and documented by the PNGIMR and approved by the Medical Research Advisory Committee of PNG (MRAC), will be followed. Information will be collected on parity, number of antenatal attendances, illness and treatment given during pregnancy and timing of routine maternal immunization with tetanus toxoid. PNGIMR will assist with transport to encourage women to attend antenatal clinics and deliver at Goroka Hospital.

# Inclusion criteria and recruitment

Infants of women intending to remain in the area for at least 2 years with a birthweight >2000 g, no acute neonatal infection and no severe congenital abnormality will be recruited. The majority of babies selected for the study will be born in hospital, but children born at home may also be included if appropriate. Note that not all mothers enrolled will have babies recruited to the study, depending on the number born in any particular week, collection of a cord blood sample and condition of the baby. Formal recruitment will begin with the routine vaccinations (BCG, OPV, HepB), which should be given within 72 hours of birth. While there will be no routine HIV testing, children of mothers known to be HIV positive will be excluded. Computer-generated lists of random numbers will be used for block randomization (see vii below).

# Immunization schedule and follow-up

Village reporters will inform study staff if a woman has delivered outside hospital. A study nurse will visit the delivery ward at Goroka Hospital at least once daily to identify newborns to enrolled women. Following recruitment, infants will be randomized to one of three equal-sized groups to receive: 1) PCV at ages 0-1-2 months, 2) PCV at ages 1-2-3 months and 3) no PCV. Birthweight and weight at each follow-up visit will be recorded. Routine infant immunizations will be given in accordance with the PNG schedule (Table 1). As PCV has not been evaluated in this population previously, the first 52 infants will be randomized to groups 2 or 3 only, and the safety data will be reviewed by an independent safety monitoring committee. Conjugate Hib vaccine (ActHib™), which is mixed with DPT, has recently been introduced into the immunization schedule in PNG, after being shown to be safe and immunogenic in this population when given at ages 1, 2 and 3 months (76). Since it is in short supply, we may have to purchase it. BCG and DPT/Hib are routinely administered in the deltoid muscle. Hepatitis B and measles will be given in the right thigh. PPV (at 9 months) and PCV will be given in the left thigh.

All immunizations will be given either in Goroka Hospital or at the PNGIMR clinic. Following immunization, children will be monitored at the clinic for 2 hours. Children will then be seen 1 and 2 days after each dose during primary immunization and on day 7 following PPV to check for local or systemic reactions. Children will be seen weekly by nursing staff to age 4 weeks. Guardians will be informed of time of appointments and transport to Goroka clinic will be arranged. If they fail to attend their appointment, we will make 3 further attempts to bring the children for routine follow-up. Guardians may choose to remove their child from the study at any time. Participants will cease to be in the study if they suffer an allergic reaction to vaccination or have inadvertently been given routine immunizations by someone other than the study nurses. All such children will continue to receive medical attention from clinic and field staff as required.

# Morbidity surveillance

Throughout the first year of life reporters will complete morbidity reports, which will be reviewed weekly by medical staff and then fortnightly to age 18 months. Parents will be encouraged and assisted to bring children in to the PNGIMR for examination and treatment if sick. Blood culture and nasal swab will be done on children with cough and raised respiratory rate (77). Children with moderate pneumonia (lower chest wall indrawing) or severe pneumonia (chest indrawing and enlarged liver or cyanosis) will be hospitalised; blood culture, nasal swab and a chest x-ray will be taken. CXRs will be read by the senior physician (I Betuela) and a sample then reviewed by a radiologist recommended by the WHO radiology group.

**iii) Safety monitoring board**

A safety monitoring board (SMB) will be established, including independent clinicians from PNG and Australia. If the board finds nothing untoward following the first block of 52 immunizations of groups 2 and 3, the trial will then be authorized to proceed including neonatal immunization (group 1). Clinical and immunogenicity data will be sent regularly to the clinical monitors. Throughout the study, all adverse events will be reported and any serious adverse events will be immediately reported to the SMB. In particular, they will examine the data for any evidence of tolerance/immune suppression in addition to serious adverse events.

# **iv) Specimen collection**

This study will involve collection of cord blood and a total of 6 blood samples over the first 18 months of life (3 samples before 6 months). Nurses at PNGIMR have extensive experience in venepuncture for culture and immunogenicity studies in young infants (5, 76). Preparation of PBMCs also allows for collection of 1.5-2ml plasma, which will be defibrinated and used for serological assays (IgG subclasses, OMP IgG) if insufficient serum is collected. This has been validated in our laboratory and defibrinated plasma is the source for the WHO pneumococcal QC sera. All specimens will reach the laboratory within 2 hours.

*Serum* (2-4ml) will be taken at birth (cord), 2, 3 and 4 months of age (initial responses to 1, 2 and 3 doses of PCV), 9 and 10 months (pre- and post-PPV booster) for memory to PCV serotypes, and for antibody persistence at 18 months.  Pnc serotype-specific IgG antibodies in serum will be measured on all samples. CPS IgG and IgG subclasses will also be measured. *Heparinised blood* (3-5ml) will be collected for T-cell cytokine studies from cord blood and at 3, 9, 10 and 18 months of age to examine development of Pnc and vaccine-specific immunity as well as overall development of Th1/Th2 immunity. This amount of blood has proven sufficient in previous studies by our group (65) for the assays proposed.

**Table 2. Outline of Serological and Cellular Assays Performed at Each Timepoint**

| Sample | Antigens | Birth* | 1  month | 2  months | 3  months | ~~4~~  months | 9  months | 10  months | 18  months |
| --- | --- | --- | --- | --- | --- | --- | --- | --- | --- |
| Serum  (2-4ml) /  Plasma | CPS IgG* | X |  | X | X | X | X | X | X |
|  | CPS IgG Avidity† | X |  | X |  | X | X | X |  |
|  | CPS IgG1 & IgG2† | X |  |  | X |  |  | X |  |
|  | Pnc OMP IgG‡ | X |  |  | X |  | X |  | X |
| PBMC  (3-5ml) | PHA, LPS/IFN, PPD, SEB | X |  |  | X |  | X | X | X |
|  | CRM_197_, DT, TT, OMP‡, HDM, RSV |  |  |  | X |  | X | X | X |
|  | Measles |  |  |  |  |  | X | X |  |
| Saliva  (1-2 ml) | CPS IgA† |  |  | X |  | X |  | X | X |
|  | Pnc OMP‡ |  | X |  | X |  | X | X | X |

* Pnc CPS types 4, 5, 6B, 9V, 7F, 14, 18C, 19F, 23F; † Pnc CPS types 6B, 7F, 14, 19F; ‡ pneumococcal OMPs PspA, PsaA, Ply

*Saliva* samples will be examined for mucosal IgA responses to vaccine and Pnc antigens. Saliva (1-2ml) will be collected using surgical eye spears, transported to the laboratory at 2-8C, centrifuged and stored at –70C. PNGIMR staff have experience in saliva collection. We have evaluated different saliva collection methods and found salivary Pnc CPS IgA collected by eye spears similar to collection by direct suction (Pomat unpublished). Eye spears are being used in a infant cohort study in Kalgoorlie, WA, and Pnc IgA assays have been successfully completed on these samples (Kyd, pers comm).

####

#### *Pernasal swabs* will be collected to determine the effect of early carriage on subsequent responses to PCV and the development of Pnc immunity and also the impact of neonatal PCV on URT carriage. Swab collection, transport and storage will employ a standardised methodology (78) which has proven to be very effective in our earlier studies in PNG (16) and Australia (11,18).

#### v) Laboratory methods

All laboratory staff will be blinded to subject treatment group.

*Bacteriology*: Standard procedures established at PNGIMR for bacterial isolation from pernasal swabs will be used, including the use of selective media and semi-quantitative assessment of colonisation density (15, 16). Samples will be stored for characterisation of respiratory pathogens other than Pnc. Serogrouping will be done on 4 Pnc colonies per plate by the Quellung reaction to detect multiple populations. Serotyping (e.g. type 6A or 6B) will be performed where appropriate by the Pneumococcal Reference Laboratory, Brisbane, through an existing relationship with PNGIMR. To examine effects of PCV on the prevalence of antibiotic-resistant isolates, antimicrobial susceptibility of Pnc will be determined by disk diffusion and suspected resistance confirmed by E-test.

*Pnc serology:* Serological testing of serum and saliva will be performed at PNGIMR under the supervision of Mr Pomat. Serotype-specific Pnc IgG serum antibodies will be measured by the standardised WHO enzyme-linked immunosorbent assay (ELISA) as previously described (21, 24) modified with preincubation of unknown sera with cell-wall polysaccharide and CPS 22F to improve assay specificity as recommended. The sera will be assayed for IgG for all seven PCV serotypes as well as non-PCV serotypes 5 and 7F that are important causes of IPD in PNG. International quality control sera (provided by D. Goldblatt, WHO Pneumococcal Serology Reference Laboratory, London) will be used to ensure consistent assay performance.

The amount of serum (or plasma) and saliva available may limit the number of additional serological investigations that can be performed. A summary of assays proposed at each time point is shown in Table 2. At least 4 representative serotypes will be selected for each assay to allow correlation between assays for serotype- specific results. These include PCV serotypes 6B, 14 and 19F (all common carriage isolates and causes of IPD in PNG) and non-PCV serotype 7F, which is the most common cause of IPD in young PNG infants (79). Antibody avidity for CPS IgG antibodies will be assessed in PNG using a modified elution ELISA with the chaotrope, sodium thiocyanate (NaSCN) as described (80) with modifications for the WHO Pnc ELISA. Avidity index is the concentration of NaSCN resulting in a 50% reduction in OD. Serotype-specific Pnc IgG_1_ and IgG_2_ subclass antibodies will be assayed in sera (or defibrinated plasma) at selected time points by standardised ELISA with 89SF reference sera as above with substitution of appropriate monoclonal enzyme conjugate. Serum IgG responses to Pnc OMPs PspA, PsaA and Ply will also be measured by ELISA as described (57).

Salivary ELISA methods that have been established (through collaboration with Dr H. Kayhty’s laboratory) to measure IgA responses to selected CPS serotypes (6B, 7F, 14, 19F) (44), and Pnc OMPs, PspA, Ply and PsaA (58), on samples as shown in Table 2. Pnc salivary IgA will be expressed as percentage of total salivary IgA. The low CPS antibody concentrations in saliva and limitations on the amount able to be collected will mean that only CPS or Pnc OMPs will be measured at time points <10 months of age (Table 2).

##

## Concomitant vaccine responses: Antibody responses to diphtheria toxoid (DT), tetanus toxoid (TT) and Hib at 4 months of age will be measured by standardised ELISA as previously described (81). Hepatitis B antibody will be measured using a routine clinical laboratory assay. Children having antibody levels that are not protective will be offered the appropriate booster immunization(s).

*Cellular immune responses:* Adaptive and innate immune functions will be assessed in children at birth, and at 3, 9, 10 and 18 months of age, employing methods in current usage in prospective cohort studies in Perth. The Holt laboratory has developed robust methods for immunoepidemiological field studies based upon on‑site collection of blood, and local processing to yield purified peripheral blood mononuclear cells (PBMC) which are immediately cryopreserved, and subsequently shipped under liquid nitrogen to a central laboratory for later assay. The T‑cell culture systems comprise bulk culture stimulation employing ≥0.5x10^6^ cells per sample, to minimise detection problems associated with low frequency of circulating responder cells.

The choice of sampling times for T-cell studies (cord, 3, 9,10 and 18 months) is based on results from our earlier studies (64, 65) and from ongoing cohort studies. Our interest in cord blood analysis is based on recent results suggesting that neonatal cytokine production profiles are predictive of immune response capacity at age 2‑4 years (82). The sample at 3 months was chosen to provide a profile of early T‑cell immunity to Pnc antigens in unvaccinated subjects, versus those who had been given 3 vaccine doses commencing at birth or delayed until one month of age. Secondarily, it will permit assessment of possible effects of PCV on postnatal maturation of overall immune competence and/or responsiveness to index vaccine components (DT, TT). The bleeds at 9 and 10 months will permit us to assess firstly the stability of PCV carrier-specific T‑cell immunity in the different groups (≥6 months after the last vaccine dose), and secondly will permit assessment of the effects of PCV on the memory T‑cell response to measles, which is the third index vaccine in the study. The final bleed at 18 months will provide data on T‑cell memory to the PCV vaccine, and a logical end-point to the section of the study involving assessment of kinetics of postnatal maturation of immune functions.

An additional important aspect of the study involves assessment of T‑cell immunity to Pnc antigens, and to this end we have obtained supplies of Pnc OMPs PspA, Ply and PsaA from Prof J. Paton, Adelaide. During the early phase of the study we will assess antibody responses to the individual Pnc OMPs, and from these results select a single Pnc OMP for the main T‑cell studies. Initial pilot experiments in our laboratories suggest PspA is the likeliest candidate. Responses to the PCV carrier protein (CRM_197_) will also be measured.

Analysis of T‑cell responses to concomitant vaccines will focus on TT and DT, and measles extract, as in current usage in T‑cell studies in our labs. This will provide information relevant to immunological safety of neonatal PCV, notably whether it modulates responses to these other vaccines e.g. via enhancement of normal postnatal maturation of Th1/Th2 functions. The latter will be independently measured via responsiveness to the polyclonal T‑cell mitogen PHA, and the innate immune system stimuli LPS/IFNγ, as in earlier studies (64, 65). An additional aspect of immunological safety monitoring will focus on potential bystander effects of reduction in carriage by PCV on respiratory mucosal homeostasis. Effects on susceptibility to viral infections such as respiratory syncytial virus (RSV), or responses to inhalant allergens, in particular house dust mite (HDM), may result in increasing respiratory tract symptoms such as wheeze and accordingly we will include RSV and HDM in the test panel. If enough cells are available, PPD (purified protein derivative of *M. tuberculosis*) and SEB (staphylococcal enterotoxin B) will be included as these provide valuable additional information on responsiveness of infants to microbial stimuli (82).

Cytokine analyses will be carried out by quantitative real-time PCR (Taqman) on cell culture pellets, or on culture supernatants by ELISA (techniques in routine use in Perth). Cytokines to be measured by ELISA will be IL‑5, IL‑6, IL‑10, IFNγ, IL‑12 and TNFα. We have determined that these cytokines are readily detectable at the protein level in the majority of infants ≥3 months of age. Cytokines to be measured by PCR (due to low production levels and/or unavailability of ELISA reagents) will be IL‑4, IL‑9, IL‑18 and IL‑23. The choice of cytokines to be measured is based upon our earlier experience with cohort studies in Perth infants. The Th2 cytokines IL‑4, IL‑5, IL‑9 and IL‑13 dominate vaccine responses in early infancy in Australian subjects, with the pattern changing towards a mixed and/or Th1‑dominated (IFNγ) response by 18 months. If “neonatal tolerance” phenomena are operative in some Pnc responses, these may be revealed as Th2‑polarised memory and hence it will be important to obtain comprehensive information on these latter cytokines. Alternatively, Th1 immunity may develop in PNG infants much more rapidly postnatally as a result of more vigorous microbial stimulation of the immune system and if so this has important implications for vaccine efficacy and safety. Accordingly we will expand the range of Th1-associated cytokines to be measured to IFNγ, TNFα, IL‑18 and IL‑23, and include IL‑12 which plays a dominant role in driving the Th1 pathway. IL‑6 and IL‑10 are important regulatory cytokines produced by both T‑cells and monocytes/macrophages, IL‑10 being of particular interest because of its potential connection with T‑regulator cell activity.

### vi) Specific scientific questions

1.  *Safety and immunogenicity of PCV under an accelerated 1-2-3 month schedule in PNG infants*

PCV has been shown to be safe and efficacious against IPD in SA infants given at 6-10-14 weeks of age (Klugman, pers comm). The standard PNG immunization schedule is at 1-2-3 months of age to achieve optimal vaccine coverage. PNG infants have higher Pnc carriage rates than reported in SA infants, which may affect immune responses to PCV. It is important that the reactogenicity and immunogenicity of PCV be established in this population at 1-2-3 months of age in comparison to control infants receiving the standard PNG infant immunizations. The response in SA infants after 1 and 2 doses of PCV at 6 and 10 weeks respectively suggests that fewer than 3 doses may be necessary in some populations (43). Therefore antibody responses to 1 and 2 doses will be examined. Immunogenicity will be primarily assessed by serotype-specific serum IgG at 2, 3 and 4 months of age, in comparison to responses in infants not receiving PCV.

2. *Safety and immunogenicity of neonatal immunization with PCV in PNG infants and comparison with early infant immunization*

Should infants be able to mount appropriate immune responses to PCV in the first week of life, then neonatal PCV should result in earlier protection against IPD due to vaccine serotypes and may provide protection against early carriage. Though neonatal responses to PCV may appear modest because of maternal antibody present, conjugate vaccines may still prime for memory (36). For our primary outcome, serum Pnc serotype-specific IgG antibody at age 2 months will be compared between neonatal PCV and control groups. This will assess whether there is a booster antibody response to the second dose of PCV in the neonatal group compared to infants given their first dose at 1 month of age and to naturally acquired antibody levels in the control group. This will determine whether neonatal PCV results in earlier protection, and monitoring will show whether safety has been compromised. We note that a safe and equally immunogenic outcome would be a more favourable response to neonatal PCV, since neonatal administration has the major logistic advantage of increasing uptake of the vaccine in the population.

*3. Induction of immunologic memory by neonatal and early infant PCV in PNG infants*

PNG infants remain at ongoing risk of IPD in the first 5 years of life. Long-term protection induced by PCV depends on the successful priming for immunologic memory so infants can mount brisk anamnestic antibody responses on subsequent challenge. All infants will receive 23-valent PPV at 9 months of age (6 months after their primary course) to evaluate the induction of memory to PCV serotypes. These serotypes in PPV are not immunogenic in PNG infants at age 9 months (except for 23F) (24) so that significant IgG responses to PCV serotypes compared to PCV-naive controls will indicate the induction of memory. This vaccine is efficacious from age 6 months and provides some cover against serotypes which are important causes of IPD in this population. Antibody avidity maturation (post-primary to post-booster immunization) will also be assessed as it is a surrogate marker for the induction of memory after conjugate vaccines (39, 80, 81) and influences the functional capacity of antibodies produced (35). Avidity maturation will be assessed by comparing changes in avidity index between 4-, 9- and 10-month samples in comparison to controls. Antibody persistence will be evaluated with blood samples at 18 months of age.

*4. The impact of neonatal versus early infant vaccination upon nasal carriage of pneumococci*

Reduction in the burden of early Pnc carriage in the critical neonatal period may have an important impact on early disease as well as the development of long-term immunity to Pnc infections. Regular carriage sampling in the first month of life will assess the impact of neonatal PCV immunization, and carriage will be assessed at regular intervals thereafter to determine persisting effects of PCV on carriage compared to controls.

*5. The impact of early colonisation with vaccine serotypes on antibody responses (systemic and mucosal) to neonatal and early infant immunization with PCV*

In the Asaro Valley 60% of infants acquired Pnc in the URT by 15 days of age. PCV serotypes are responsible for 59% of carriage isolates in the first 3 months of life in PNG (16). Swabs will be collected at weekly intervals during the first month to identify children with early (≤ 2 weeks) colonisation. This will allow evaluation of the effects of early colonisation on systemic antibody responses to subsequent PCV immunization. Effects of time of colonisation and PCV on mucosal immunity (salivary IgA responses to Pnc CPS and OMPs) will also be assessed .

6. The relationship between the capacity to respond to Pnc antigens and developmental factors controlling postnatal maturation of immune competence

(a) Development of natural immunity to Pnc antigens

Humoral immunity will be assessed as serum IgG and salivary IgA responses to CPS and Pnc OMPs at regular intervals, and cellular immunity as in vitro Th1/Th2 cytokine production. In this initial assessment of natural immunity, we will focus on the study group who do not receive PCV. Serological assays for measuring serum and salivary antibody responses to Pnc CPS and OMPs established in our laboratory will provide detailed information on the ontogeny of humoral Pnc systemic and mucosal immunity in this high-risk population.

Our forerunner T-cell studies on a cohort of 130 subjects (64, 65) have demonstrated that the kinetics of postnatal cytokine response capacity is highly variable amongst Australian infants, and in particular Th1 response capacity is slow to develop over the first 18 months of life. Moreover, development of this function appears to be rate-limiting in relation to competence to respond to vaccines, and may be similarly related to competence to resist infection (65). Comparable data of this nature are not available from similar-sized cohorts of infants in third world countries, where the overall levels of microbial exposure are much higher. It is now accepted that microbial exposure during infancy constitutes the principal stimulus for postnatal maturation of Th1 function and it is possible that Th1 functions in PNG infants may mature much faster, due to the higher levels of environmental microbial stimulation. We propose to resolve this important question via analysis of generalised (and Pnc‑specific) immunity in the PNG cohort, compared with data from the Perth cohort (64, 65).

(b) Development of PCV-induced T‑cell immunity to Pnc antigens

Based on experience from studies on Australian infants, we will seek to ascertain whether quantitative and qualitative aspects of vaccine-induced Pnc CPS and carrier protein‑specific immunity are directly related to the underlying capacity to transcribe cytokine genes in polyclonal-induced responses, and/or whether vaccine response capacity is related to kinetics of maturation of cells of the innate immune system (in particular, IL‑12 responses). Studies on Perth children also suggest that aspects of the functional capacity of cord blood mononuclear responses are predictive of subsequent immunological reactivity during infancy (82), and on this basis the proposed PNG analyses will also include cord blood functions and how these relate to subsequent postnatal maturation of immune competence including responsiveness to PCV.

Our previous findings indicated a strong Th2‑bias in early vaccine responses, and relative instability in Th1 memory up to 18 months of age (64, 65). In relation to vaccine design, this argues for considering the use of stronger (Th1-trophic) adjuvants to boost vaccine responses in this age range. However, the parallel situation with respect to vaccine responsiveness in infants in a third world setting is largely undefined, and different strategies may need to be considered for improvement of vaccine efficacy in such populations. Information relevant to this important issue can be readily obtained in this study by comparing data on tetanus-specific T‑cell responses in PNG infants to those found in the earlier Perth study. These data will also be employed in relation to assessment of vaccine safety (see 8 below).

(c) Colonisation with Pnc

We will additionally test the possibility that variations in the functional competence at birth of cells of the innate and adaptive arms of the immune system may influence susceptibility to colonisation with Pnc during the immediate postnatal period. A variety of evidence from our group and others derived from studies on cord blood cells (83) suggests that delayed/attenuated functional maturation of Th1‑associated effector mechanisms during foetal life may result in some individuals being in a transient state of “developmental immunosuppression” in early infancy, relative to the population at large. This subgroup will potentially respond to environmental antigens less efficiently than the population average. We will assess polyclonal T‑cell function and innate immune function in cord blood cells from PNG infants, and relate these data to subsequent patterns of Pnc colonisation and manifestations of natural and vaccine-induced immunity to Pnc, to ascertain whether these outcomes are influenced by immune status at birth.

*7. Early carriage of Pnc as a potential cause of impaired responses to vaccine and non-vaccine Pnc antigens*

We will seek to define the relationship between the timing of nasal colonisation with Pnc and (i) the kinetics of acquisition of natural and vaccine-induced Pnc‑specific immunity, and (ii) the Th1/Th2 balance within these responses. Of particular interest will be the possibility that early colonisation with Pnc in the neonatal period may induce a form of “tolerance” associated with priming of Th2-polarised immunity to Pnc‑antigen(s), which may in turn modulate host capacity to develop natural or vaccine-induced immunity to Pnc.

8. Assessment of immunological safety of neonatal immunization with PCV in PNG infants

(a) Possible interference with immune responses to concomitant vaccines (DTP and measles)

Similar to concerns with early onset Pnc carriage, neonatal immunisation with PCV may result in preferential Th2 priming for the PCV carrier protein CRM_197_ which may affect subsequent responses to the virtually identical DT component of DTP vaccine. The potential for non-specific interference with responses to two additional “index” vaccines, the TT component of DTP vaccine which elicits a mixed Th0‑like response in infants which includes a strong Th2 component (64, 65), and the measles vaccine which elicits a response with a stronger Th1 component, will also be assessed. T‑cell cytokine responses to all these vaccines will be measured in the three study groups, to ascertain in particular whether neonatal PCV modulates the Th1/Th2 balance in host responses. Antibody responses to concomitant TT, Hib (PRP-TT) and DT will be measured at 4 months of age to ensure that there has been no interference with humoral immune responses. In this population all children achieve protective anti-tetanus and anti-diphtheria toxin antibodies after 3 doses of ActHib™ (76).

*(b)* *Non-specific interference with normal maturation of the immune system, in particular Th1 immunity*

Given the important role of microbial stimulation in driving the postnatal maturation of immune function(s), it is theoretically possible that PCV, particularly if given in the neonatal period, may contribute to this drive and hence accelerate the normal maturation process. This represents a possible safety issue, as (for example) precocious postnatal maturation of Th1 function may pose potential risks for inflammatory disease. Alternatively, PCV may promote non-specific Th2 immunity and delay Th1 maturation resulting in increased susceptibility to infectious and allergic diseases. Accordingly, specific analyses will be performed to contrast the kinetics of postnatal maturation of Th1/Th2 response capacity in groups with and without neonatal PCV.

*9. The impact of PCV on respiratory morbidity in PNG infants in the first year of life*

Asaro Valley infants on average suffer 3 ALRI episodes in the first year of life (84). To ensure the safety of neonatal or early PCV and any potential benefits of PCV on respiratory morbidity, there will be close monitoring of infants by trained field staff throughout the study using established methods. Though this proposed study is complete in itself, we intend to continue to follow up participants after completion of the study at age 18 months to ensure ongoing safety in terms of respiratory morbidity, hospitalisation and death.

**vii) Sample size requirements**

**Urban Rural n**

Group #2 #3 #1 #2 #3 #1 312

Block 1 13 13 13 13 52

Block 2 13 13 26 13 13 26 104

Blocks 3-4 26 26 26 26 26 26 156

The block randomisation schema is set out above. The sample size requirements were based on the primary questions and assumed 80% power and a 5% significance level. Distributions of published Pnc vaccine responses (21, 43, 44) indicate a standard deviation of serotype-specific IgG concentration of around 1 on the natural logarithmic scale, at all ages. For serious adverse reactions in Block 1, 26 subjects will be allocated to the post-neonatal PCV group 2 and 26 to the control group 3. This comparison will allow proportions of adverse events of e.g. 10% in the control group versus 48% in the treated group, or 5% vs 40%. If no serious adverse events occur in the first 26 treated children, it is likely that the true adverse event rate is 10% or less (and similarly in Block 2 for neonatal PCV group 1). Given 70% seroprotection (>0.5μg/ml) after 1 dose of PCV at age 6 weeks (43), the sample size of 100 per group (min. 90 evaluable) allows this proportion to be estimated with 95% CI of 60-79% and will have 80% power at the 5% significance level to detect a 20% difference from group 1 (i.e. 90%) or group 3 (i.e. 50%).

For ALRI hospital admissions, assuming a rate of 0.0425 per child month under 1 year, a 50% decrease in either vaccinated group compared with controls is detectable. We can detect differences in carriage rates between the groups from 1 month onwards of say 40% down to 22% or lower.

# **viii) Data analysis**

The principal analyses of efficacy and safety will be based on t-tests and chi-square tests. Further analyses examining the association of different factors such as number in household, housing type, town/country etc with response will use multiple regression methods: linear when IgG or cytokine level is the response, logistic when IgA, carriage or cytokine detection is the response, Cox when time to hospital admission or onset of carriage is the response, or Poisson when admission rate is the response.

Comparison of cytokine levels between the different groups at different times will employ the same basic methods; however, further analyses of immune competence will be slightly more complex in that the profile of within-person changes over time in antibody and cytokine levels will form the major response and explanatory variables. Thus 2-level models allowing for different levels of variability within and between children will be used, comparing in particular both level and rate of change of these measures. These regression models will also include terms for vaccination status as well as the other confounders such as crowding, sex etc.

***5. Contributions of the partners and added value of the proposed partnership***

Pneumonia is a major health priority of the PNG Health Plan. Although PNGIMR targets its research program to reflect the National Health needs, it has been difficult in recent years to obtain the funding required to maintain a strong respiratory disease program. This project, in addition to addressing a very important research question, will help PNGIMR rejuvenate its research program on respiratory diseases. PNGIMR has a long history of conducting vaccine trials and has established immunological and bacteriological facilities. This collaboration combines researchers with extensive experience in field research, epidemiology, bacteriology and immunology in PNG with state-of-the-art immunology and molecular techniques to address fundamental health issues in a high-risk population. The project will provide a critical mass of activity and training to provide a framework on which to build an ongoing program and to seek diversified funding opportunities to ensure sustainability. ICHR and the Department of Paediatrics, University of Western Australia (UDP) are members of the PNGIMR Buttressing Coalition. After 17 years as epidemiologist in PNG (where she was also training officer for PNGIMR), D Lehmann has ongoing collaborations with PNGIMR to complete studies of ARI and the impact of vaccination on childhood mortality and is working on otitis media and respiratory bacteria in Australia. Training of PNGIMR staff in Perth in immunological techniques is ongoing with P Richmond and P Holt. Professor Holt is a pre-eminent cellular immunologist who has made major contributions to the understanding of immune responses to allergens and the normal maturation of the immune system. He has extended this to the generation of vaccine-specific cellular immunity in early life. P Richmond is a paediatric immunologist with extensive experience in vaccine trials and serological assays. J Reeder is the director of the IMR based in Goroka. He is a microbiologist with the field experience and ability to successfully coordinate the laboratory and field components in PNG. All collaborators will be fully involved in the study design and analysis, to which N de Klerk will have an essential statistical input.

Field work will be conducted by staff at PNGIMR with assistance in re-establishing surveillance by DL. Initial sample processing, bacteriology, Pnc serology and cryopreservation of PBMC will be conducted in PNG. A team of Papua New Guineans have extensive experience in carrying out demographic and morbidity surveillance as well as safety and immunogenicity studies. W Pomat, a PNGIMR staff member for the past 15 years, is currently investigating mucosal immune responses to Pnc vaccines in Perth as part of a PhD program and will be able to establish assays in the PNGIMR laboratories on return as well as assist in training other staff. PBMC samples will be sent to Perth for T-cell studies. PNGIMR staff will be trained in these techniques in Perth to facilitate future studies in this area.

1. **Description of planned training activities in the developing country**

Two PNGIMR laboratory staff will come to Prof. Holt’s laboratory in Perth for 3 months for training in specimen collection processing and PBMC cryopreservation. A PNGIMR Scientific Officer, who is incidentally from the study area, will be based in Perth for 2 years for training and to participate in the T-cell studies and will enrol in a Masters degree program. Nursing staff will be trained in taking of cord blood and infant blood and saliva samples.

PNGIMR staff member Mr W Pomat is currently undertaking PhD studies in Perth under the supervision of the project collaborators and will return to PNG to take part in the project. He will contribute to on-the-job training of technical staff there and help to build a critical mass of PNG scientists trained in immunology. The project will also strengthen training and exchange activities with clinical staff at the Goroka Hospital, in particular Dr I Betuela and the paediatricians. This will be reinforced by a visit of P Richmond to PNG. The time spent by D Lehmann in PNG will be important not only for establishing the field aspects of the study but also for restoring contact with the field staff she trained in the past and initiating the training of new staff.

- 1. **Expected outcomes/health benefits/policy implications**

Invasive and mucosal Pnc infections in early infancy are a major global health burden. The potential advantages of neonatal immunization include high vaccination coverage, protection against very early Pnc carriage, reduction in mortality and morbidity due to pneumonia and IPD in early infancy, and enhancement of subsequent protection by PPV. Benefits are also likely to occur for the prevention of OM and its complications in high-risk populations. Detailed studies are essential to ensure “immunological safety” of this approach and to further our understanding of mechanisms of actions of bacterial conjugate vaccines. Public concerns over increasing numbers of vaccines overloading children’s immune system mean that in-depth immunological studies are needed globally to provide a solid basis for ensuring the safety of immunization in infants and to address these safety concerns explicitly. This study will provide basic immunological understanding to inform the epidemiological investigation of the immune responses achieved and the safety and possible non-specific benefits of immunization in a tropical setting. Furthermore, the results of this study will be particularly relevant for the prevention of Pnc disease in the ‘fourth world’ – in young indigenous children of first world countries who suffer similarly high rates of Pnc disease and early URT carriage.

Australian investigators will gain expertise in field research in non-industrialised countries, which will help enhance collaboration with groups in other countries in the region. Through detailed investigation of immune responses in infants exposed to numerous pathogens from a very young age, this collaboration will complement studies conducted in Australia towards understanding the development of the immune system in infancy.

This proposed study will have important implications for the development of pneumococcal immunization policy in PNG and other non-industrialised countries. If neonatal immunization with PCV is found to be safe, the collaborators will be in a strong position to proceed to an efficacy study and work towards establishing the most effective and cost-efficient schedules for PCV and the associated use of PPV.

**References**

1. Lancet. The world's forgotten children. Lancet 2003;361:1.

2. Shann F, Steinhoff MC. Vaccines for children in rich and poor countries. Lancet 1999;354(Suppl II):7-11.

3. Butler JC, Crengle S, Cheek JE, Leach AJ, Lennon D, O'Brien KL, Santosham M. Emerging infectious diseases among indigenous peoples. Emerg Infect Dis 2001;7:554-5.

4. Torzillo PJ, Hanna JN, Morey F, Gratten M, Dixon J, Erlich J. Invasive pneumococcal disease in central Australia. Med J Aust 1995;162:182-6.

5. The WHO Young Infant Study Group. Bacterial etiology of serious infections in young infants in developing countries: results of a multicenter study. Pediatr Infect Dis J 1999;18(10 Suppl):S17-22.

6. Kakazo M, Lehmann D, Coakley K, Gratten H, Saleu G, Taime J, Riley ID, Alpers MP. Mortality rates and the utilization of health services during terminal illness in the Asaro Valley, Eastern Highlands Province, Papua New Guinea. PNG Med J 1999;42:13-26.

7. Barker J, Gratten M, Riley I, Lehmann D, Montgomery J, Kajoi M, Gratten H, Smith D, Marshall TF, Alpers MP. Pneumonia in children in the Eastern Highlands of Papua New Guinea: a bacteriologic study of patients selected by standard clinical criteria. J Infect Dis 1989;159:348-52.

8. Duke T, Michael A, Mgone J, Frank D, Wal T, Sehuko R. Etiology of child mortality in Goroka, Papua New Guinea: a prospective two-year study. Bull World Health Organ 2002;80:16-25.

9. Lehmann D, Yeka W, Rongap T, Javati A, Saleu G, Clegg A, Michael A, Lupiwa T, Omena M, Alpers MP. Aetiology and clinical signs of bacterial meningitis in children admitted to Goroka Base Hospital, Papua New Guinea, 1989-1992. Ann Trop Paediatr 1999;19:21-32.

10. Michael A, Wal T, Duke T, Yohannes M, Kakazo M, Sehuko R, Siba P, Lehmann D, Poigeno G, Mgone J, Frank D, Oswin G, Kurubi J. Multi-drug resistance in paediatric meningitis patients admitted to the Goroka Base Hospital. Abstract in Program and Abstracts of the 38th Annual Symposium of the Medical Society of Papua New Guinea, Alotau, Milne Bay Province, 9-13 Sep 2002:43-44.

11. Leach A, Boswell JB, Asche V, Nienhuys TG, Mathews JD. Bacterial colonization of the nasopharynx predicts very early onset and persistence of otitis media in Australian Abriginal infants. Pediatr Infect Dis J 1994;13:983-9.

12. Riley ID, Tarr PI, Andrews M, Pfeiffer M, Howard R, Challands P, Jennison G. Immunisation with a polyvalent pneumococcal vaccine. Reduction of adult respiratory mortality in a New Guinea highlands community. Lancet 1977;1:1338-41.

13. Smith T, Lehmann D, Montgomery J, Gratten M, Riley ID, Alpers MP. Acquisition and invasiveness of different serotypes of Streptococcus pneumoniae in young children. Epidemiol Infect 1993;111:27-39.

14. Gratten M, Gratten H, Poli A, Carrad E, Raymer M, Koki G. Colonisation of Haemophilus influenzae and Streptococcus pneumoniae in the upper respiratory tract of neonates in Papua New Guinea: primary acquisition, duration of carriage, and relationship to carriage in mothers. Biol Neonate 1986;50:114-20.

15. Gratten M, Montgomery J. The bacteriology of acute pneumonia and meningitis in children in Papua New Guinea: assumptions, facts and technical strategies. PNG Med J 1991;34:185-98.

16. Montgomery JM, Lehmann D, Smith T, Michael A, Joseph B, Lupiwa T, Coakley C, Spooner V, Best B, Riley ID, Michael MP. Bacterial colonization of the upper respiratory tract and its association with acute lower respiratory tract infections in Highland children of Papua New Guinea. Rev Infect Dis 1990;12(Suppl 8):S1006-16.

17. Gray BM, Converse GM, III, Dillon HC, Jr. Epidemiologic studies of Streptococcus pneumoniae in infants: acquisition carriage, and infection during the first 24 months of life. J Infect Dis 1980;142:923-33.

18. Lehmann D, Bowman J, Riley TV, Murphy D, Elsbury D, Dorizzi L, Monck R. Nasopharyngeal bacterial carriage in young Aboriginal and non-Aboriginal children in an arid zone of Western Australia. Abstract in Program and Abstracts of the Third International Symposium on Pneumococci and Pneumococcal Diseases, Anchorage, 6-8 May 2002:50.

19. Faden H, Duffy L, Wasielewski R, Wolf J, Krystofik D, Tung Y, Tonawada/Williamsville Pediatrics. Relationship between nasopharyngeal colonization and the development of otitis media in children. J Infect Dis 1997;175:1440-45.

20. Hausdorff WP, Siber G, Paradiso PR. Geographical differences in invasive pneumococcal disease rates and serotype frequency in young children. Lancet 2001;357:950-2.

21. Lehmann D, Pomat WS, Combs B, Dyke T, Alpers MP. Maternal immunization with pneumococcal polysaccharide vaccine in the highlands of Papua New Guinea. Vaccine 2002;20:1837-45.

22. Gray BM, Dillon HC, Jr. Epidemiological studies of Streptococcus pneumoniae in infants: antibody to types 3, 6, 14, and 23 in the first two years of life. J Infect Dis 1988;158:948-55.

23. Soininen A, Pursiainen H, Kilpi T, Kayhty H. Natural development of antibodies to pneumococcal capsular polysaccharides depends on the serotype: association with pneumococcal carriage and acute otitis media in young children. J Infect Dis 2001;184:569-76.

24. Pomat WS, Lehmann D, Sanders RC, Lewis DJ, Wilson J, Rogers S, Dyke T, Alpers MP. Immunoglobulin G antibody responses to polyvalent pneumococcal vaccine in children in the highlands of Papua New Guinea. Infect Immun 1994;62:1848-53.

25. Witt CS, Pomat W, Lehmann D, Alpers MP. Antibodies to pneumococcal polysaccharides in pneumonia and response to pneumococcal vaccination in young children in Papua New Guinea. Clin Exp Immunol 1991;83:219-24.

26. Simell B, Kilpi T, Rapola S, Virva J, Lahdenkari M, Kayhty H. Anti-PsaA, -PspA and -Ply in saliva of children and the risk of pneumococcal acute otitis media. Abstract in Program and Abstracts of the Third International Symposium on Pneumococci and Pneumococcal Diseases, Anchorage, 6-8 May 2002:82.

27. Makela PH, Karma P, Sipila M, Pukander J, Leinonen M. Possibilities of preventing otitis media by vaccination. Scand J Infect Dis Suppl 1983;39:34-8.

28. Mogdasy MC, Camou T, Fajardo C, Hortal M. Colonizing and invasive strains of Streptococcus pneumoniae in Uruguayan children: type distribution and patterns of antibiotic resistance. Pediatr Infect Dis J 1992;11:648-52.

29. Capeding MR, Sombrero LT, Lucero MG, Saniel MC. Serotype distribution and antimicrobial resistance of invasive Streptococcus pneumoniae isolates in Filipino children. J Infect Dis 1994;169:479-80.

30. O'Dempsey TJ, McArdle TF, Lloyd-Evans N, Baldeh I, Lawrence BE, Secka O, Greenwood B. Pneumococcal disease among children in a rural area of west Africa. Pediatr Infect Dis J 1996;15:431-7.

31. Riley ID, Lehmann D, Alpers MP, Marshall TF, Gratten H, Smith D. Pneumococcal vaccine prevents death from acute lower-respiratory-tract infections in Papua New Guinean children. Lancet 1986;2:877-81.

32. Lehmann D, Marshall TF, Riley ID, Alpers MP. Effect of pneumococcal vaccine on morbidity from acute lower respiratory tract infections in Papua New Guinean children. Ann Trop Paediatr 1991;11:247-57.

33. Lehmann D, Vail J, Firth MJ, de Klerk NH, Alpers MP. Impact of routine immunisations on childhood survival in Tari, Southern Highlands Province, Papua New Guinea. submitted 2002.

34. Wright PF, Sell SH, Vaughn WK, Andrews C, McConnell KB, Schiffman G. Clinical studies of pneumococcal vaccines in infants. II. Efficacy and effect on nasopharyngeal carriage. Rev Infect Dis 1981;3 Suppl:S108-12.

35. Usinger WR, Lucas AH. Avidity as a determinant of the protective efficacy of human antibodies to pneumococcal capsular polysaccharides. Infect Immun 1999;67:2366-70.

36. Siegrist C. Neonatal and early life vaccinology. Vaccine 2001;19:3331-46.

37. Black S, Shinefield H, Fireman B, Lewis E, Ray P, Hansen JR, Elvin L, Ensor KM, Hackell J, Siber G, Malinoski F, Madore D, Chang I, Kohberger R, Watson W, Austrian R, Edwards K. Efficacy, safety and immunogenicity of heptavalent pneumococcal conjugate vaccine in children. Northern California Kaiser Permanente Vaccine Study Center Group. Pediatr Infect Dis J 2000;19:187-95.

38. Dagan R, Eskola J, Leclerc C, Leroy O. Reduced response to multiple vaccines sharing common protein epitopes that are administered simultaneously to infants. Infect Immun 1998;66:2093-8.

39. Anttila M, Eskola J, Ahman H, Kayhty H. Differences in the avidity of antibodies evoked by four different pneumococcal conjugate vaccines in early childhood. Vaccine 1999;17:1970-7.

40. Wuorimaa T, Kayhty H, Eskola J, Bloigu A, Leroy O, Surcel HM. Activation of cell-mediated immunity following immunization with pneumococcal conjugate or polysaccharide vaccine. Scand J Immunol 2001;53:422-8.

41. Black SB, Shinefield HR, Ling S, Hansen J, Fireman B, Spring D, Noyes J, Lewis E, Ray P, Lee J, Hackell J. Effectiveness of heptavalent pneumococcal conjugate vaccine in children younger than five years of age for prevention of pneumonia. Pediatr Infect Dis J 2002;21:810-5.

42. Mbelle N, Huebner RE, Wasas AD, Kimura A, Chang I, Klugman KP. Immunogenicity and impact on nasopharyngeal carriage of a nonavalent pneumococcal conjugate vaccine. J Infect Dis 1999;180:1171-6.

43. Huebner RE, Mbelle N, Forrest B, Madore DV, Klugman KP. Immunogenicity after one, two or three doses and impact on the antibody response to coadministered antigens of a nonavalent pneumococcal conjugate vaccine in infants of Soweto, South Africa. Pediatr Infect Dis J 2002;21:1004-7.

44. Nurkka A, Ahman H, Korkeila M, Jantti V, Kayhty H, Eskola J. Serum and salivary anti-capsular antibodies in infants and children immunized with the heptavalent pneumococcal conjugate vaccine. Pediatr Infect Dis J 2001;20:25-33.

45. Eskola J, Kilpi T, Palmu A, Jokinen J, Haapakoski J, Herva E, Takala A, Kayhty H, Karma P, Kohberger R, Siber G, Makela PH. Efficacy of a pneumococcal conjugate vaccine against acute otitis media. N Engl J Med 2001;344:403-9.

46. O'Brien KL, Steinhoff MC, Edwards K, Keyserling H, Thoms ML, Madore D. Immunologic priming of young children by pneumococcal glycoprotein conjugate, but not polysaccharide, vaccines. Pediatr Infect Dis J 1996;15:425-30.

47. Kurikka S, Kayhty H, Peltola H, Saarinen L, Eskola J, Makela PH. Neonatal immunization: response to Haemophilus influenzae type b- tetanus toxoid conjugate vaccine. Pediatrics 1995;95:815-22.

48. Lieberman JM, Greenberg DP, Wong VK, Partridge S, Chang SJ, Chiu CY, Ward JI. Effect of neonatal immunization with diphtheria and tetanus toxoids on antibody responses to Haemophilus influenzae type b conjugate vaccines. J Pediatr 1995;126:198-205.

49. Ward JI, Bulkow L, Wainwright RV, Chang SJ. Immune tolerance and lack of booster responses to Haemophilus influenzae (Hib) conjugate vaccination in infants immunized beginning at birth. Abstract in Program and Abstracts of the 32nd Interscience Conference on Antimicrobial Agents and Chemotherapy (ICAAC), Anaheim, CA 1992:984.

50. World Health Organization. WHO meeting on maternal and neonatal pneumococcal immunization. Wkly Epidemiol Rec 1998;73:187-8.

51. Kristensen I, Aaby P, Jensen H. Routine vaccinations and child survival: follow up study in Guinea-Bissau, West Africa. Bmj 2000;321:1435-8.

52. Briles DE, Hollingshead S, Brooks-Walter A, Nabors GS, Ferguson L, Schilling M, Gravenstein S, Braun P, King J, Swift A. The potential to use PspA and other pneumococcal proteins to elicit protection against pneumococcal infection. Vaccine 2000;18:1707-11.

53. Obaro SK, Adegbola RA, Tharpe JA, Ades EW, McAdam KP, Carlone G, Sampson JS. Pneumococcal surface adhesin A antibody concentration in serum and nasopharyngeal carriage of Streptococcus pneumoniae in young African infants. Vaccine 2000;19:411-2.

54. Musher DM, Phan HM, Baughn RE. Protection against bacteremic pneumococcal infection by antibody to pneumolysin. J Infect Dis 2001;183:827-30.

55. McCool TL, Cate TR, Moy G, Weiser JN. The immune response to pneumococcal proteins during experimental human carriage. J Exp Med 2002;195:359-65.

56. Virolainen A, Russell W, Crain MJ, Rapola S, Kayhty H, Briles DE. Human antibodies to pneumococcal surface protein A in health and disease. Pediatr Infect Dis J 2000;19:134-8.

57. Rapola S, Jantti V, Haikala R, Syrjanen R, Carlone GM, Sampson JS, Briles DE, Paton JC, Takala AK, Kilpi TM, Kayhty H. Natural development of antibodies to pneumococcal surface protein A, pneumococcal surface adhesin A, and pneumolysin in relation to pneumococcal carriage and acute otitis media. J Infect Dis 2000;182:1146-52.

58. Simell B, Korkeila M, Pursiainen H, Kilpi TM, Kayhty H. Pneumococcal carriage and otitis media induce salivary antibodies to pneumococcal surface adhesin a, pneumolysin, and pneumococcal surface protein a in children. J Infect Dis 2001;183:887-96.

59. Wegmann TG, Lin H, Guilbert L, Mosmann TR. Bidirectional cytokine interactions in the maternal-fetal relationshiip: is successful pregnancy a Th2 phenomenon? Immunol Today 1993;14:353-6.

60. Ridge JP, Fuchs EJ, Matzinger P. Neonatal tolerance revisited: turning on newborn T cells with dendritic cells. Science 1996;271:1723-26.

61. Lee SM, Suen Y, Chang L, Bruner V, Qian J, Indes J, Knoppel E, van de Ven C, Cairo MS. Decreased interleukin-12 (IL-12) from activated cord versus adult peripheral blood mononuclear cells and upregulation of interferon-g, natural killer, and lymphokine-activated killer activity by IL-12 in cord blood mononuclear cells. Blood 1996;88:945-54.

62. Upham JW, Lee PT, Holt BJ, Heaton T, Prescott SL, Sharp MJ, Sly PD, Holt PG. Development of interleukin-12-producing capacity throughout childhood. Infect Immun 2002;70:6583-8.

63. White GP, Watt PM, Holt BJ, Holt PG. Differential patterns of methylation of the IFNg promoter at CpG and non-CpG sites underlie differences in IFNg gene expression between human neonatal and adult CD45RO^-^ T-cells. J Immunol 2002;168:2820-7.

64. Rowe J, Macaubas C, Monger TM, Holt BJ, Harvey J, Poolman JT, Sly PD, Holt PG. Antigen-specific responses to diphtheria-tetanus-acellular pertussis vaccine in human infants are initially Th2 polarized. Infect Immun 2000;68:3873-7.

65. Rowe J, Macaubas C, Monger T, Holt BJ, Harvey J, Poolman JT, Loh R, Sly PD, Holt PG. Heterogeneity in diphtheria-tetanus-acellular pertussis vaccine-specific cellular immunity during infancy: relationship to variations in the kinetics of postnatal maturation of systemic Th1 function. J Infect Dis 2001;184:80-8.

66. Reen DJ. Activation and functional capacity of human neonatal CD4 T cells. Vaccine 1998;16:1401-8.

67. Marchant A, Goetghebuer T, Ota M, Wolfe I, Ceesay SJ, De Groote D, Corrah T, Bennett S, Wheeler J, Huygen K, Aaby P, McAdam KP, Newport MJ. Newborns develop a Th1-type immune response to Mycobacterium bovis bacillus Calmette-Guerin vaccination. J Immunol 1999;163:2249-55.

68. Aberle JH, Aberle SW, Dworzak MN, Mandl CW, Rebhandl W, Vollnhofer G, Kundi M, Popow-Kraupp T. Reduced interferon-g expression in peripheral blood mononuclear cells of infants with severe respiratory syncytial virus disease. Am J Respir Crit Care Med 1999;160:1263-8.

69. Bont L, Heijnen CJ, Kavelaars A, van Aalderen WMC, Brus F, Draaisma JMT, Pekelharing-Berghuis M, van Diemen-Steenvoorde RAAM, Kimpen JLL. Local interferon-g levels during respiratory syncytial virus lower respiratory tract infection are associated with disease severity. J Infect Dis 2001;184:355-8.

70. Renzi PM, Turgeon JP, Marcotte JE, Drblik SP, Bérubé D, Gagnon MF, Spier S. Reduced interferon-g production in infants with bronchiolitis and asthma. Am J Respir Crit Care Med 1999;159:1417-22.

71. Blanco-Quirós A, González H, Arranz E, Lapeña S. Decreased interleukin-12 levels in umbilical cord blood in children who developed acute bronchiolitis. Pediatr Pulmonol 1999;28:175-80.

72. Adkins B, Du R-Q. Newborn mice develop balanced Th1/Th2 primary effector responses *in vivo* but are biased to Th2 secondary responses. J Immunol 1998;160:4217-24.

73. Culley FJ, Pollott J, Openshaw PJ. Age at first viral infection determines the pattern of T cell-mediated disease during reinfection in adulthood. J Exp Med 2002;196:1381-86.

74. Holt PG, Sly PD. Interactions between RSV infection, asthma, and atopy: unraveling the complexities. J Exp Med 2002;196:1271-75.

75. Duchmann R, Schmitt E, Knolle P, Meyer zum Büschenfelde K-H, Neurath M. Tolerance towards resident intestinal flora in mice is abrogated in experimental colitis and restored by treatment with interleukin-10 or antibodies to interleukin-12. Eur J Immunol 1996;26:934-938.

76. Lehmann D, Kakazo M, Saleu G, Taime J, Javati A, Namuigi P, Alpers MP, Wegmuller B, Zellmeyer M, Furer E, Que JU, Herzog C. Safety and immunogenicity of two Haemophilus influenzae type b polysaccharide-tetanus toxoid conjugate vaccines (PRP-T) given with diphtheria-tetanus-pertussis vaccine to young Papua New Guinean children. PNG Med J 2001;44:6-16.

77. Papua New Guinea Department of Health. Standard treatment of common illnesses of children in Papua New Guinea. A manual for nurses, health extension officers and doctors. Sixth ed. Port Moresby: Department of Health, 1993.

78. O'Brien KL, Bronsdon MA, Dagan R, Yagupsky P, Janco J, Elliott J, Whitney CG, Yang YH, Robinson LG, Schwartz B, Carlone GM. Evaluation of a medium (STGG) for transport and optimal recovery of Streptococcus pneumoniae from nasopharyngeal secretions collected during field studies. J Clin Microbiol 2001;39:1021-4.

79. Lehmann D, Gratten M, Montgomery J. Susceptibility of pneumococcal carriage isolates to penicillin provides a conservative estimate of susceptibility of invasive pneumococci. Pediatr Infect Dis J 1997;16:297-305.

80. Richmond P, Borrow R, Goldblatt D, Findlow J, Martin S, Morris R, Cartwright K, Miller E. Ability of 3 different meningococcal C conjugate vaccines to induce immunologic memory after a single dose in UK toddlers. J Infect Dis 2001;183:160-3.

81. Goldblatt D, Richmond P, Millard E, Thornton C, Miller E. The induction of immunologic memory after vaccination with Haemophilus influenzae type b conjugate and acellular pertussis-containing diphtheria, tetanus, and pertussis vaccine combination. J Infect Dis 1999;180:538-41.

82. Sharp MJ, Rowe J, Kusel M, Sly PD, Holt PG. Specific patterns of responsiveness to microbial antigens SEB and PPD by cord blood mononuclear cells are predictive of risk for development of Atopic Dermatitis. Clin Exp Allergy; In press.

83. Holt PG, Jones CA. The development of the immune system during pregnancy and early life. Allergy 2000;55:688-97.

84. Smith TA, Lehmann D, Coakley C, Spooner V, Alpers MP. Relationships between growth and acute lower-respiratory infections in children aged less than 5 y in a highland population of Papua New Guinea. Am J Clin Nutr 1991;53:963-70.
